# From hazard to risk prioritization: a case study to predict drug-induced cholestasis using physiologically based kinetic modeling

**DOI:** 10.1007/s00204-024-03775-6

**Published:** 2024-05-17

**Authors:** Véronique M. P. de Bruijn, Ivonne M. C. M. Rietjens

**Affiliations:** Division of Toxicology, Wageningen University and Research, Wageningen, The Netherlands

**Keywords:** Bile acids and salts, Physiologically based kinetic (PBK) modeling, Cholestasis, Adverse outcome pathway

## Abstract

Cholestasis is characterized by hepatic accumulation of bile acids. Clinical manifestation of cholestasis only occurs in a small proportion of exposed individuals. The present study aims to develop a new approach methodology (NAM) to predict drug-induced cholestasis as a result of drug-induced hepatic bile acid efflux inhibition and the resulting bile acid accumulation. To this end, hepatic concentrations of a panel of drugs were predicted by a generic physiologically based kinetic (PBK) drug model. Their effects on hepatic bile acid efflux were incorporated in a PBK model for bile acids. The predicted bile acid accumulation was used as a measure for a drug’s cholestatic potency. The selected drugs were known to inhibit hepatic bile acid efflux in an assay with primary suspension-cultured hepatocytes and classified as common, rare, or no for cholestasis incidence. Common cholestasis drugs included were atorvastatin, chlorpromazine, cyclosporine, glimepiride, ketoconazole, and ritonavir. The cholestasis incidence of the drugs appeared not to be adequately predicted by their *K*_i_ for inhibition of hepatic bile acid efflux, but rather by the AUC of the PBK model predicted internal hepatic drug concentration at therapeutic dose level above this *K*_i_. People with slower drug clearance, a larger bile acid pool, reduced bile salt export pump (BSEP) abundance, or given higher than therapeutic dose levels were predicted to be at higher risk to develop drug-induced cholestasis. The results provide a proof-of-principle of using a PBK-based NAM for cholestasis risk prioritization as a result of transporter inhibition and identification of individual risk factors.

**Supplementary Information:**

The online version contains supplementary material available at 10.1007/s00204-024-03775-6.

## Introduction

Drug-Induced Liver Injury (DILI) is the most frequent cause for drug development discontinuation and DILI incidence is expected to increase, because of an increased dependency on drugs by the aging population (Walker et al. 2020). DILI is classified in a hepatocellular, cholestatic, and a mixed type (Yu et al. 2017), where cholestatic DILI is characterized among others by a disrupted bile flow. While the DILI incidence is very low, with < 1 in 1000 to 1 in 100,000 users (Kaplowitz 2005; Fontana et al. 2023), the clinical outcome to the individual is devastating. Cholestatic liver injury constitutes 20–40% of DILI cases (Sundaram and Bjornsson 2017), which underscores the critical necessity for robust tools to identify potentially cholestatic drug candidates early in the drug-discovery phase and identification of sensitive individuals. Besides drugs, certain food additives, dietary supplements, biocides, and industrial chemicals can induce cholestasis (Vilas-Boas et al. 2019, 2020). The present study aims to develop a new approach methodology (NAM) to predict drug-induced cholestasis as a result of drug-induced hepatic bile acid efflux inhibition and the resulting bile acid accumulation.

In recent years, substantial attention has been directed toward the development of Adverse Outcome Pathways (AOPs) as a conceptual framework for toxicological risk assessment. AOPs consist of a sequence that encompasses a Molecular Initiating Event (MIE), one or more Key Events (KEs), and an Adverse Outcome (AO), collectively representing responses spanning various tiers of biological complexity (Ankley et al. 2010). The fundamental principle underlying AOPs is that the MIE(s) combined with a select set of KEs can elucidate and forecast a toxicological response. Notably, these identified MIE(s) and KEs are amenable to exploration through NAMs, thereby facilitating a mechanism-centered, animal-free approach to assessing the safety of chemicals (Leist et al. 2017). Inhibition of the hepatic bile salt export pump (BSEP) is considered as MIE in the AOP of cholestasis. BSEP-inhibition leads to intrahepatic bile acid accumulation and subsequent toxicity (Vinken et al. 2013). A recent case study elucidated that two azole fungicides (propiconazole and tebuconazole) inhibited BSEP-mediated bile acid transport and affected several nuclear receptors (Knebel et al. 2022) which is in line with the AOP. Propiconazole and tebuconazole did not induce cholestasis in standard rodent in vivo bioassays (Heise et al. 2015; Schmidt et al. 2016; Nielsen et al. 2012), although for clinically used azole fungicides, cholestasis has been reported in the European database of suspected adverse drug reaction (ADR) reports (www.adrreports.eu; accessed on the 21st of August 2023). Knebel et al. explain the discrepancy between the outcomes of their in vitro testing strategy and the rodent assays by the fact that the intrahepatic propiconazole and tebuconazole concentrations in the in vivo bioassays were too low to induce BSEP inhibition (Schmidt et al. 2016). Hence, the in vitro testing strategy probably successfully revealed use of propiconazole and tebuconazole as a hazard for cholestasis, but for a risk assessment, it is important to consider organ concentrations. Organ concentrations can be derived from studies with laboratory animals or in an animal-free approach using physiologically based kinetic (PBK) modeling.

To predict drug-induced cholestasis, the drug PBK model predicting internal hepatic concentrations in humans of a series of selected drugs was incorporated in a bile acid PBK model describing the synthesis, circulation, and excretion of the most abundant bile acid in human serum, glycochenodeoxycholic acid (GCDCA) (Bathena et al. 2013). Conjugated bile acids, like GCDCA, are typically transported by carrier-mediated transport, while unconjugated bile acids are mainly transported over the liver membranes via passive processes and thus unlikely to be affected by transporter inhibition (Notenboom et al. 2018). Simulating only one bile acid enabled us to keep the model complexity to a minimum, making the model easier to interpret and minimizing the risk of overfitting. The selected drugs are all known to inhibit hepatic bile acid efflux but are classified as common, rare, or no for their incidence of inducing cholestasis. The time-dependent drug-induced intrahepatic GCDCA accumulation was determined and compared to the inhibitory constant (*K*_i_) of the drug for hepatic bile acid efflux inhibition as a measure for cholestatic potency. This newly defined approach also enabled prediction of the chances on developing cholestasis in people with slower clearance of the drug, a larger bile acid pool, reduced BSEP abundance, or given higher than therapeutic dose levels. Thus, this modeling approach serves a proof-of-concept to predict drug-induced cholestasis based on NAMs.

## Methods

### Selection and classification of drugs

The criteria for the inclusion of a drug in our panel were (a) causally linked to the development of DILI by the U.S. Food and Drug Administration (FDA) (Chen et al. 2016), (b) oral administration in clinical practice, (c) able to inhibit bile acid efflux with the half inhibitory concentration (IC_50_) for bile acid efflux available from an assay with human suspension-cultured hepatocytes and the IC_50_ being < 100 µM (Zhang et al. 2016), (d) the reported DILI being not immune-mediated, and (e) two or more in vivo pharmacokinetic studies available in the literature to validate the drug PBK model-based predictions for plasma concentrations. The maximal concentration used for the IC_50_ determination by Zhang et al. (2016) was 100 µM. This resulted in a final inclusion of 18 drugs, i.e., atorvastatin, bicalutamide, bosentan, chlorpromazine, cyclosporine, deferasirox, fluoxetine, flutamide, glimepiride, haloperidol, lovastatin, ketoconazole, pioglitazone, ritonavir, rosiglitazone, saquinavir, trazodone, and troglitazone. Flutamide, lovastatin, and saquinavir were excluded from further simulations based on the results from the generic PBK model, as described in “Glycochenodeoxycholic acid PBK model” section. The drugs resulted in different types of DILI. The LiverTox® database classified chlorpromazine, cyclosporine, and ritonavir as cholestatic DILI, and the remaining drugs were classified as hepatocellular/mixed DILI (http://LiverTox.nih.gov; last accessed on the 24th of August 2023). For atorvastatin, the LiverTox® database reported two cases of mixed, one of cholestatic and one of hepatocellular DILI. Glimepiride and haloperidol were not present in the LiverTox® database. The cholestasis incidence of the 15 remaining drugs was evaluated based on the European database of suspected adverse drug reaction (ADR) reports (www.adrreports.eu; last accessed on the 24th of August 2023). The following adverse reactions were considered cholestatic: cholestasis, cholestatic liver injury, cholestatic jaundice, and cholestatic hepatitis. The incidence of cholestasis was classified by us, using the European ADR database as follows: common (> 0.5% of ADR cholestatic), rare (0.3–0.5% of ADR cholestatic), and no (< 0.3% of ADR cholestatic). The threshold for common incidence was set to 0.5% of ADR to ensure that the drugs chlorpromazine, cyclosporine, and ritonavir, which were identified as cholestatic in the LiverTox® database, were classified as common causes of cholestasis. The 0.3% threshold was set artificially to account for background cholestasis incidence. Additional drugs classified as common for induction of cholestasis according to our classification were: bosentan, ketoconazole, atorvastatin, and glimepiride. Riede et al. (2017) reviewed cholestasis incidence of several drugs based on cohort and retrospective studies. In line with our classification, Riede et al. (2017) classified cyclosporine cholestasis incidence as common and rosiglitazone as not cholestatic, but ketoconazole and atorvastatin cholestasis incidences were classified as rare in contrast to our classification system. No explanation was found for the discrepancy between atorvastatin and ketoconazole cholestasis incidence classification  by these authors and the ADR database, hence, we used our classification system based on the European ADR database. Bosentan was classified as rare or common depending on the dose in the review of Riede et al. (2017). At therapeutic dose level, i.e., 125 mg twice a day, bosentan was considered a rare cause of cholestasis. Since the drugs were evaluated at their therapeutic dose level in the current study, bosentan-induced cholestasis incidence was classified as rare. Troglitazone was banned from the market and therefore not in the European ADR database, and also not in the review by Riede et al. (2017). A review of cohort studies indicated that troglitazone causes hepatocellular liver injury, with rare instances of mixed or cholestatic liver injury (Chojkier 2005). Troglitazone cholestasis incidence was therefore classified as rare. Maximal prescribed daily dosage was used for simulations and obtained from the supplier’s prescription information.

### Generic PBK models for drugs

A generic PBK model was used to predict the hepatic concentrations of the selected drugs at therapeutic dose level and above. These concentrations were subsequently used to predict the inhibitory effect on hepatic bile acid efflux and resulting bile acid accumulation using a coupled bile acid PBK model (see “Glycochenodeoxycholic acid PBK model” section).

The generic drug PBK models were adapted from Punt et al. (2022). Briefly, the PBK models consisted of compartments for lung, adipose, bone, brain, heart, intestine, liver, kidney, muscle, skin, spleen, and arterial and venous blood. Different compared to Punt et al. (2022), a blood:plasma ratio of 0.55 was used for acidic compounds (1-hematocrit), and 1 for neutral or basic compounds (Cubitt et al. 2009). Physicochemical properties (pKa, log*P*, log*D*, topological surface area, and molecular weight) of the drugs were predicted using Chemicalize, https://chemicalize.com/ developed by ChemAxon (http://www.chemaxon.com). The physicochemical properties were subsequently used to predict tissue:plasma partition coefficients (Berezhkovskiy 2004; Rodgers and Rowland 2006), absorption rate constants, and fractions absorbed (Hou et al. 2004).

As part of this study, several in vitro and in silico methods were evaluated to derive the tissue:plasma partition coefficients, hepatic intrinsic clearance, and fraction unbound in plasma (*F*_up_) (Table 1). In more detail, for the tissue:plasma partition coefficients, values derived using the in silico methods of Rodgers and Rowland (2006) and Berezhkovskiy (2004) were compared. For the fraction unbound (*F*_up_), both the in silico method of Lobell and Sivarajah (2003) and in vitro rapid equilibrium dialysis data using human plasma were evaluated. Clearance data were derived from in vitro hepatocyte studies or the pkCSM in silico tool (Pires et al. 2015). It should be noted that in vitro the hepatic intrinsic clearance was measured, while pkCSM predicted the total clearance, i.e., a combination of hepatic and renal clearance. Where possible, in vitro intrinsic hepatic clearance (CL_int_) and *F*_up_ data were obtained from the high-throughput toxicokinetic (httk) database (Pearce et al. 2017); alternatively, a literature search in Scopus was conducted to obtain the in vitro CL_int_ (Supplementary Table S1). In vitro CL_int_ was determined using fresh or cryopreserved primary human hepatocytes (PHH) cultured in a monolayer or suspension. The CL_int_ was determined based on substrate depletion or metabolite formation. The incubation medium did not contain any serum and was optimized for hepatocyte function, maintaining physiological temperature and pH.

**Table 1:** Input parameters for the generic PBK model

Process	Parameter(s)	Method	Number of drugs	References
Intestinal uptake	Ka, Fa	QSAR based on the topological surface area	18	Hou et al. (2004)
Physicochemical parameters	pKa, logP, logD, topocological surface area, molecular weight	Chemicalize (in silico)	18	https://chemicalize.com/ developed by ChemAxon
Tissue:plasma partition coefficients	Kpad, Kpbo, Kpbr, Kpgu, Kphe, Kpki, Kpli, Kplu, Kpmu, Kpsk, Kpsp	In silico	18	Rodgers and Rowlands (2006)
		In silico	18	Berezhkovskiy (2004)
Hepatic intrinsic clearance	CL_int_	(Cryopreserved) primary human hepatocytes (in vitro*)*	15	Data derived from the httk package (Pearce et al. 2017) or other publications (see Table S1)
Total clearance	CL_tot_	pkCSM (in silico)	18	Pires et al. (2015)
Fraction unbound plasma	*F*_up_	Equilibrium dialysis (in vitro)	18	Data derived from the httk package (Pearce et al. 2017), or other publications (Hahn et al. 1973; Zaghloul et al. 1987)
		In silico	18	Lobell and Sivarajah (2003)
Blood:plasma ratio	BP	Acidic drugs: 0.55 (1-hematocrit) Neutral or basic drugs: 1	18	Cubitt et al. (2009)

Ka: absorption rate constant, Fa: fraction absorbed, Kpad, Kpbo, Kpbr, Kpgu, Kphe, Kpki, Kpli, Kplu, Kpmu, Kpsk, and Kpsp are tissue:plasma partition coefficients for adipose tissue, bone, brain, gut, heart, kidney, liver, lung, muscle, skin, and spleen, respectively

*QSAR* quantitative structure activity relationship

Corrections for non-specific binding of the compounds to the hepatocytes in vitro were applied based on the calculation method of Kilford et al. (2008). The in vitro and in silico clearance data were scaled to the in vivo situation based on a hepatocellularity of 117.5 × 10^6^ hepatocytes per gram liver (Barter et al. 2007) and a liver weight of 1470 g, or 70 kg body weight, respectively, see Eqs. 1 and 2

1 $${\text{CL}}_{{{\text{int}} ,{\text{in}}\;{\text{vivo}}}} = {\text{CL}}_{{{\text{int}} ,{\text{in}}\;{\text{vitro}}}} \times {\text{Hep}} \times {\text{Vli}} \times 60 \times 10^{ - 6} ,$$

2 $${\text{CL}}_{{{\text{tot}},{\text{in}}\;{\text{vivo}}}} = {\text{CL}}_{{{\text{tot}},{\text{in}}\;{\text{silico}}}} \times {\text{BW }} \times 60 \times 10^{ - 3} ,$$

where in Eq. 1, CL_int,in vivo_ is the intrinsic clearance in vivo in L min^−1^ entire liver^−1^, CL_int,in vitro_ the hepatic intrinsic clearance in vitro in µL min^−1^ 10^–6^ hepatocytes, Hep the hepatocellularity in 10^6^ hepatocytes/g liver, and Vli the weight of the liver in grams. A factor of 10^–^^6^ L µL^−1^ and 60 min h^−1^ was applied to convert the CL_int_ to units applicable to the PBK model. Equation 2 describes the in silico-to-in vivo scaling of the total clearance (CL_tot_). CL_tot,in vivo_ is the total clearance in vivo L min^−1^ entire liver^−1^, CL_tot,in silico_ the total clearance as predicted by pkCSM in mL min^−1^ kg body weight^−1^, and BW the body weight in kg. Here, factors of 10^–3^ L mL^−1^ and 60 min h^−1^ were applied to convert the CL_tot_ to units applicable to the PBK model.

A literature search was performed to compile a dataset on human in vivo drug plasma peak concentrations (*C*_max_) after a single oral dose of the selected drugs. The following keywords were used in Scopus: ((TITLE (“drug name”) AND ALL (bioavailability OR pharmacokinetics OR kinetics)) AND (( human OR man OR volunteer OR subject)) AND (Cmax OR “c max” OR “maximal concentration” OR “maximum concentration” OR “peak concentration”)). The studies that were identified for each drug were subsequently filtered to exclude (1) results obtained for specific patient groups like patients with renal impairment or gastric by-pass, (2) studies with children, and (3) studies using slow or extended-release formulations. At least two *C*_max_ values per drug were collated from different doses and/or studies. The dose (normalized to bodyweight), *C*_max_ and references were gathered in the file “Invivo.xlsx” in the Github repository.^1^ The final dataset included 115 studies and 179 sets of dose and corresponding *C*_max_ values for 18 drugs. A default bodyweight of 70 kg was assumed if the bodyweight was not reported in the study. *C*_max_ values were averaged if experiments were performed under both fasted and fed conditions. Most studies reported peak concentrations in plasma, but if blood concentrations were reported, they were converted to plasma concentrations based on the blood:plasma ratio (see Table 1). The studies included a variable number of human volunteers per group with an arithmetic mean of 20 (range: min. 5–max. 89).

The predicted plasma *C*_max_ was compared with the observed plasma *C*_max_ as obtained from the meta-analysis. The ratio predicted:observed *C*_max_ was calculated for each study and/or dose. This resulted in a number of ratios per compound. The median of this ratio was calculated per compound, and for further simulations, the combination of input parameters that gave a median ratio predicted:observed *C*_max_ closest to 1 was selected. Only the drugs of with a median ratio predicted:observed *C*_max_ within tenfold were used for further analysis (*n* = 15). An overview of the metabolizing enzymes or transporters involved in the kinetics of the drugs was made to find explanations for over- or underpredictions. The information about the involved enzymes and transporters was obtained from literature (Wishart et al. 2018; Elsby et al. 2012; Cockshott 2004; Hebert 1997; Klatt et al. 2011; Treiber et al. 2007).

### Glycochenodeoxycholic acid PBK model

**Fig. 1:**
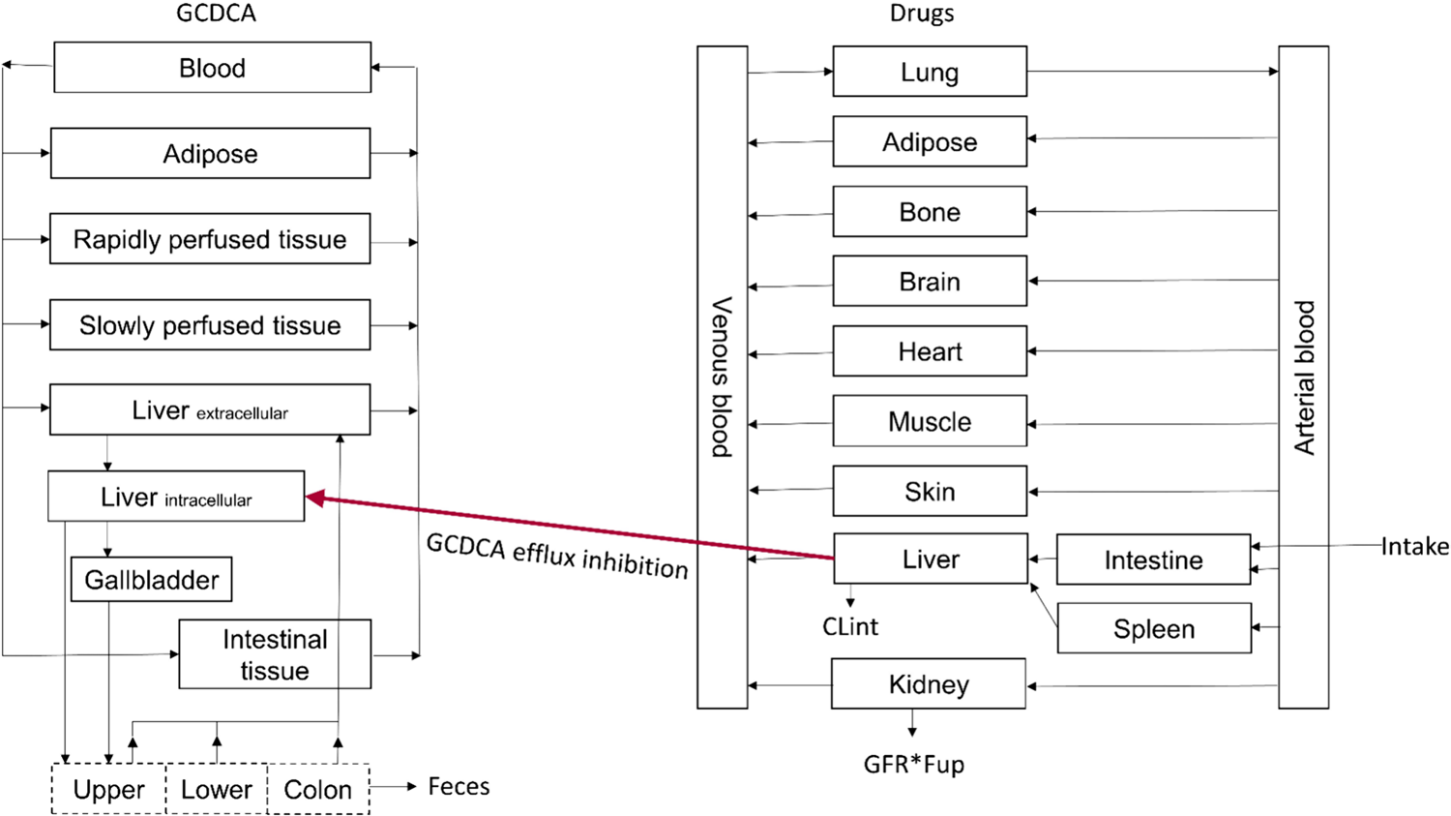
Conceptual model for the PBK modeling of bile acid homeostasis and the influence on this homeostasis by drugs. Conceptual model for GCDCA PBK model was taken from de Bruijn et al. (2023), and conceptual model for generic drugs PBK models was taken from Punt et al. (2022). CL_int_: intrinsic clearance; *F*_up_: fraction unbound plasma; GCDCA: glycochenodeoxycholic acid; GFR: glomerular filtration rate

A PBK model describing synthesis, circulation and excretion of bile acids in healthy individuals was based on our previous work (de Bruijn et al. 2022, 2023). The conceptual model is presented in Fig. 1. The enterohepatic circulation was modeled as a circulation of GCDCA between the liver (extracellular and intracellular), gallbladder, and intestine. The intestinal uptake and the hepatic uptake and efflux were described using carrier-mediated transport processes, i.e., ASBT, NTCP, or BSEP-mediated, respectively. The NTCP-mediated hepatic uptake of GCDCA was modeled permeability-limited as described in our previous work (de Bruijn et al. 2023). The kinetic parameters for ASBT-mediated transport were obtained using Caco-2 cells cultured on permeable cell culture inserts and scaled from the in vitro to in vivo situation as described in our previous work (de Bruijn et al. 2023). GCDCA de novo synthesis in the liver was set equal to its excretion via the feces. GCDCA was actively transported from the liver to the common bile duct by BSEP following Michaelis–Menten kinetics. The BSEP-mediated efflux of GCDCA was described by Eq. 3

3 $$E = \frac{{V_{{\max ,{\text{BSEP}}}} \times \left[ {{\text{Cliveriw}}} \right]}}{{K_{{m,{\text{BSEP}}}} + \left[ {{\text{Cliveriw}}} \right]}},$$

where *E* is the BSEP-mediated efflux in µmol/h, *V*_max_ is the maximum efflux rate of GCDCA in blood in µmol/entire liver/h, [Cliveriw] the free concentration of bile acids in intracellular water in liver in µmol/L and *K*_m,BSEP_ the Michaelis–Menten constant in µmol/L for BSEP-mediated GCDCA efflux. The *V*_max_ and *K*_m_ for BSEP-mediated transport of GCDCA were obtained from a vesicular transport assay in a baculovirus-infected Sf9 system (Kis et al. 2009).

The differential model equations were encoded and solved using the deSolve package version 1.32 in R version 4.1.0 (Soetaert and Petzoldt 2010; R Core Team 2022). The model code can be found in the Github repository (see Footnote 1).

### Inhibitory effect of drugs on hepatic bile acid efflux

The hepatic-free concentration of the within tenfold predicted drugs at the maximal prescribed daily therapeutic dosage was used to simulate their inhibitory effect on hepatic GCDCA efflux. The conceptual PBK model for the drugs was combined with the PBK model for GCDCA as displayed in Fig. 1. The concentrations required to reduce hepatic bile acid efflux by 50% (IC_50_) were derived from a study using suspension-cultured primary human hepatocytes (PHHs) (Zhang et al. 2016) and corrected for in vitro non-specific binding (Kilford et al. 2008). The IC_50_ values obtained using PHHs were for all drugs except pioglitazone lower than the IC_50_ values obtained using BSEP-transfected membrane vesicles (Supplementary Table S2). Therefore, the results obtained from suspension-cultured PHHs were used for further simulations as a worst-case estimate. Membrane vesicles and suspension-cultured PHHs provide different insights in hepatic bile acid efflux. PHHs are known to have a physiologically relevant expression of several transporters and metabolizing enzymes, rather than the exclusive BSEP expression in membrane vesicles. Hence, in contrast to membrane vesicles, the drug-induced inhibitory effect on bile acid efflux from primary suspension-cultured human hepatocytes is not necessarily caused by an exclusive inhibition of BSEP-mediated transport, but could also be caused by inhibition of the uptake, basolateral efflux by MRP3/4 or the conjugation process. Nevertheless, the net drug-induced bile acid efflux inhibition was incorporated in the equation for BSEP-mediated efflux. The PHHs were commercially obtained and pooled from 10 donors, including 5 males and 5 males aged 17–65. Cells were cultured in suspension at a density of 0.25 × 10^6^ cells/mL in the presence of 10 µM cholic acid and various drug concentrations. Upon incubation, the PHHs conjugated the cholic acid with a glycine group resulting in glycocholic acid. After incubation, intra- and extracellular bile acid concentrations were quantified using LC–MS/MS. In the absence of specific data on the effects of all the selected drugs on bile acid efflux for GCDCA, glycocholic acid efflux was used as a surrogate for all drugs (Zhang et al. 2016). Glycocholic acid showed greater inhibition (lower IC_50_) in assays with PHHs than GCDCA and was considered as a worst-case estimate of GCDCA efflux (Chothe et al. 2021; Yucha et al. 2017). Competitive inhibition was assumed, as this is the typical mode of drug-transporter-inhibition (Kenna et al. 2018). The following formula to calculate *K*_i_ applies for competitive inhibitors like the drugs considered here (Eq. 4) (Yung-Chi and Prusoff 1973):

4 $$K_{{\text{i}}} = \frac{{{\text{IC}}_{50} }}{{1 + \frac{\left[ S \right]}{{K_{{\text{m}}} }}}},$$

where *K*_i_ is the inhibitory constant in µM, IC_50_ the half maximum inhibitory concentration of the drug in µmol/L, [S] the substrate concentration in µM, and *K*_m_ the Michaelis–Menten constant in µM.

Subsequently, the inhibitory effect of the drugs on GCDCA efflux was incorporated in the PBK model equation describing BSEP-mediated efflux. In line with competitive inhibition, the *K*_m,BSEP_ in the corresponding Michaelis–Menten reaction (Eq. 3) was modified to the apparent *K*_m_ (*K*_m,BSEP,app_) as follows (Eq. 5):

5 $$K_{{{\text{m}},{\text{BSEP}},{\text{app}}}} = K_{{{\text{m}},{\text{BSEP}}}} \left( {1 + \frac{\left[ I \right]}{{K_{{{\text{i}} }} }}} \right),$$

where *K*_m,BSEP,app_ is the apparent Michaelis–Menten constant in µM, [*I*] the unbound hepatic concentration of the inhibitor (= drug) in µM and *K*_i_ the inhibitory constant in µM.

The dose-metric used to evaluate intracellular GCDCA accumulation was the area under the curve (AUC), because it has been acknowledged AUC is the most relevant for endpoints that are influenced by total dose over time resulting in an accumulation (Rietjens et al. 2019).

### Simulating sensitive individuals

The developed PBK approach was also employed to evaluate drug effects on intrahepatic GCDCA accumulation in sensitive individuals. For these studies, cyclosporine was selected as the model drug, because the maximal prescribed daily therapeutic dose resulted in intrahepatic concentrations equivalent to the *K*_i_ for bile acid efflux inhibition, facilitating detection of changes in the bile acid accumulation. In our previous work, we established that an over 1.5-fold increased total bile acid pool size posed an individual at risk for intrahepatic bile acid accumulation as a result of BSEP-inhibition (de Bruijn et al. 2022). Furthermore, a low BSEP abundance was identified as a potential risk factor for the development of cholestasis. Therefore, as an example, the effects of cyclosporine on intrahepatic accumulation were simulated for (a) a reference individual, (b) an individual with a 1.5-fold increased total bile acid pool size compared to the reference individual, (c) an individual with low hepatic BSEP abundance, or (d) an individual with an increased pool size and a low BSEP abundance. The low and reference BSEP abundances were derived from a meta-analysis of hepatic transporter abundances in healthy Caucasians (Burt et al. 2016). The in vitro to in vivo extrapolation of *V*_max_ was based on the BSEP abundance. A lower BSEP abundance thus resulted in a lower in vivo *V*_max_. The reference individual had a BSEP protein abundance of 0.84 pmol BSEP protein per a million hepatocytes. Low BSEP abundance was set to the reported mean minus three times the standard deviation and amounted to 0.23 pmol BSEP protein per million hepatocytes.

### Sensitivity analysis

To assess the influence of the parameters on the model outcome, a sensitivity analysis was performed for the plasma *C*_max_ of the drugs and the intrahepatic GCDCA levels. The drug’s doses were set to 1 mg/kg body weight. For plasma *C*_max_, all potential combinations of input parameters were evaluated. The normalized sensitivity coefficients (NSC) for hepatic GCDCA levels were calculated using the combination of input parameters giving the drug’s *C*_max_ in closest agreement with the in vivo data (Supplementary Table S3). Based on the method reported by Evans and Andersen (2000), the normalized sensitivity coefficients (NSCs) for the model parameters were calculated as follows:

6 $${\text{NSC}} = \frac{{C^{\prime} - C}}{{P^{\prime} - P}} \times P/C,$$

where *C* indicates the initial value of the model output, and *C*′ indicates the modified value of the model output resulting from an increase in the parameter value. *P* indicates the initial parameter value and *P*′ indicates the modified parameter value after a 5% increase of its value, keeping all other parameters at their original value.

## Results

### Tissue:plasma partitioning has a major impact on *C*_max_ for acidic drugs

**Fig. 2:**
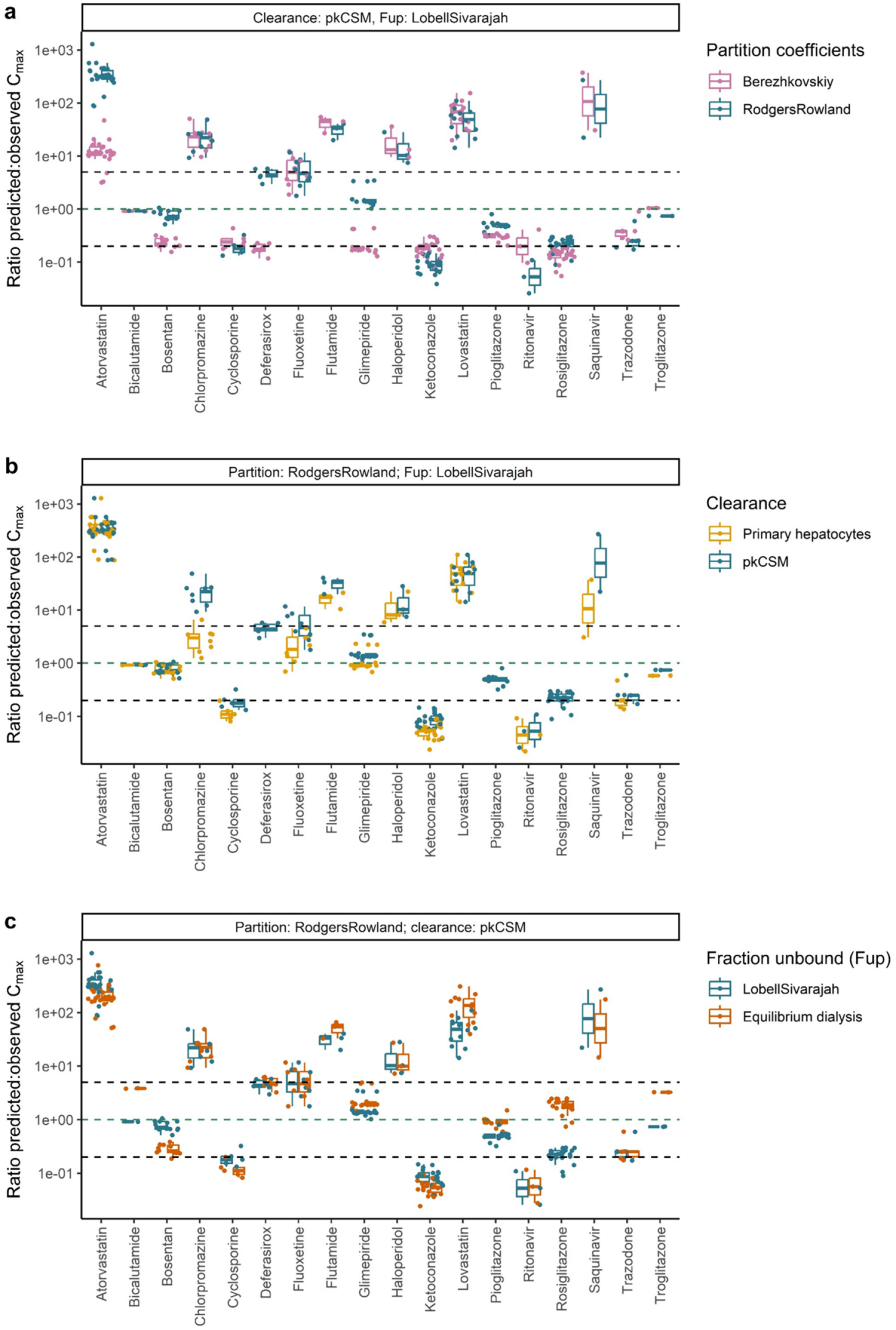
Ratio predicted:observed *C*_max_ for the 18 selected drugs using different methods to obtain the PBK model input parameters for **a** partition coefficients, **b** clearance, and **c** fraction unbound (*F*_up_), while the other methods to obtain the input parameters where as indicated in the box above **a**–**c**. Green dashed line: ratio predicted:observed = 1; lower and upper black dashed lines: ratio predicted:observed = 0.2 or 5, respectively

Figure 2 visualizes the effect of altering the method for obtaining the partition coefficients, the intrinsic clearance, or *F*_up_ parameter, while keeping the methods for defining the other generic drug PBK model parameters unchanged. For 13 out of 18 drugs, the *C*_max_ predictions by the generic model were within fivefold of the observed data for at least one combination of the drug PBK model input parameters. The predicted *C*_max_ of atorvastatin and haloperidol were more than fivefold but less than tenfold overpredicted. The *C*_max_ of saquinavir, flutamide, and lovastatin were more than tenfold overpredicted for all combinations of input parameters (Supplementary Material Figure S2). The largest effect on *C*_max_ was observed for the method used to define the partition coefficients (Fig. 2A). For drugs with pKa < 6, i.e., atorvastatin, bosentan, deferasirox, and glimepiride, calculating the partition coefficients by the method of Berezhkovskiy resulted in an over fivefold lower prediction for the *C*_max_ as compared to the results obtained with the method of Rodgers and Rowland. The *F*_up_ and clearance methods had smaller effects on plasma *C*_max_. Intrinsic total clearance predicted by pkCSM resulted in slightly higher or similar *C*_max_ values as intrinsic hepatic clearance determined using (cryopreserved) primary hepatocytes (Fig. 2B). Supported by this close agreement between the two sets of *C*_max_ predictions, pkCSM was employed to predict clearance for rosiglitazone, pioglitazone, and deferasirox for which no in vitro clearance data were available. This approach resulted in *C*_max_ predictions within fivefold compared to the observed pharmacokinetic data.

**Fig. 3:**
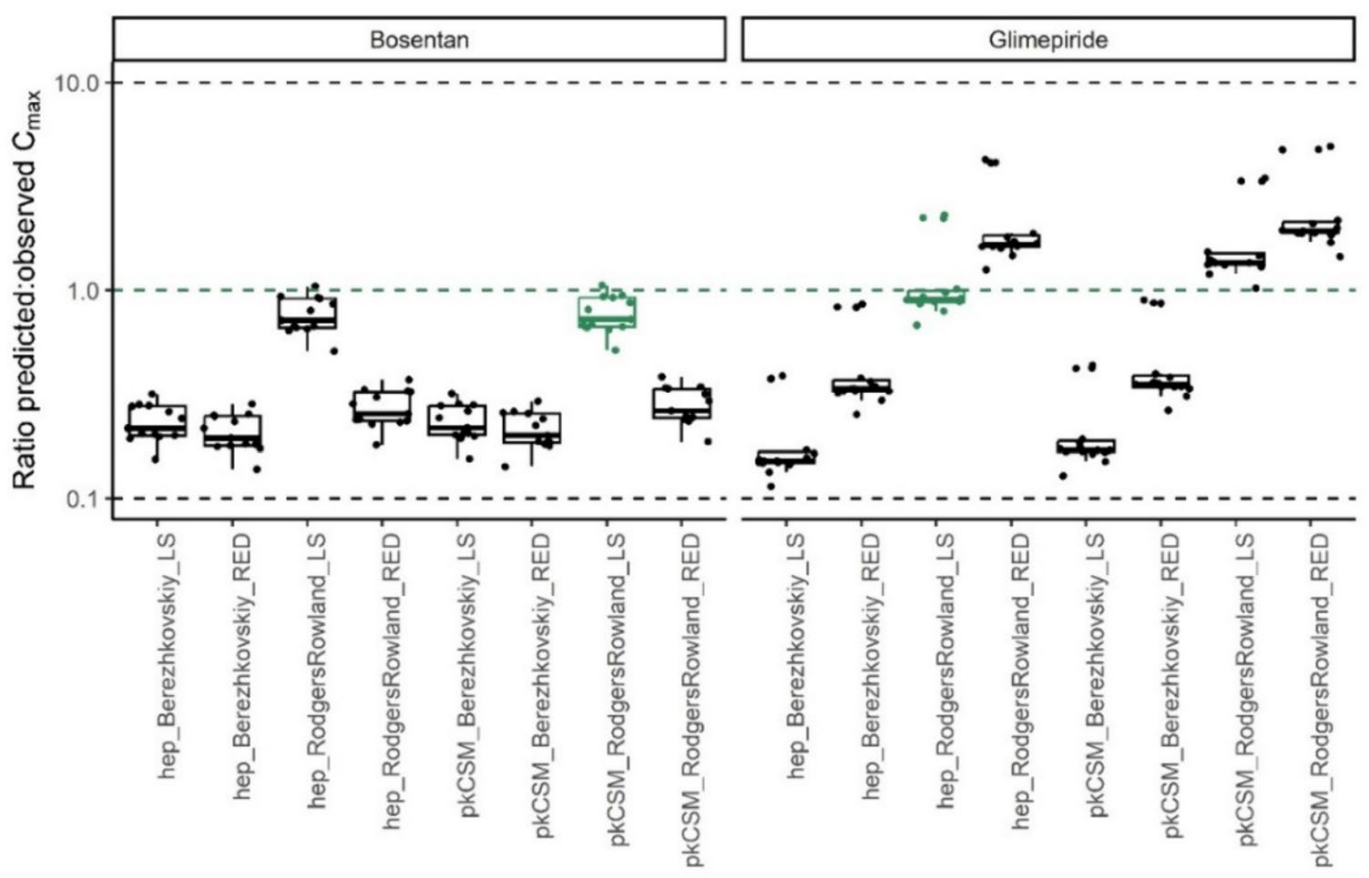
Ratio predicted:observed *C*_max_ for bosentan and glimepiride using eight different combinations of PBK model input parameters. The green box indicates that this combination of input parameters resulted in a median ratio predicted:observed closest to 1. The combination in green is used for further simulations. Clearance: hep = primary hepatocytes, pkCSM = in silico clearance, partition coefficients: Berezhkovskiy or Rodgers and Rowlands, fraction unbound in plasma: LS = Lobell Sivarajah, RED = rapid equilibrium dialysis. Green dashed line: ratio predicted:observed = 1, lower and upper black dashed lines: ratio predicted:observed = 0.1 or 10, respectively

The hepatic free concentration of the drugs was predicted using the combination of input parameters giving the *C*_max_ in closest agreement with the in vivo data. Exemplary Fig. 3 indicates which combination of input parameters gave the most accurate prediction for the *C*_max_ of bosentan and glimepiride. The closest agreement with in vivo data was achieved for bosentan and glimepiride when the *C*_max_ values were predicted using the method of Rodgers and Rowland for tissue partitioning and the method of Lobell and Sivarajah for calculation of *F*_up_. *C*_max_ was predicted best when determining the clearance by the in silico tool pkCSM and primary hepatocytes for bosentan and glimepiride, respectively. These comparisons were made for all 18 drugs and are displayed in 6.5 Supplementary file Figure S1. In case two methods resulted in exactly the same median ratio predicted: observed, the in silico methods were chosen for intrinsic clearance and *F*_up_. Supplementary Table S3 provides a tabular overview of the methods chosen for prediction of the parameters for further simulations. Flutamide, saquinavir, and lovastatin were excluded from further predictions because of the > tenfold overprediction of their* C*_max_. Even though *C*_max_ predictions above fivefold but within tenfold of the observed *C*_max_ are not very precise, they are still considered relevant (Punt et al. 2021).

### Kinetic processes involved in the kinetics of the drugs

**Fig. 4:**
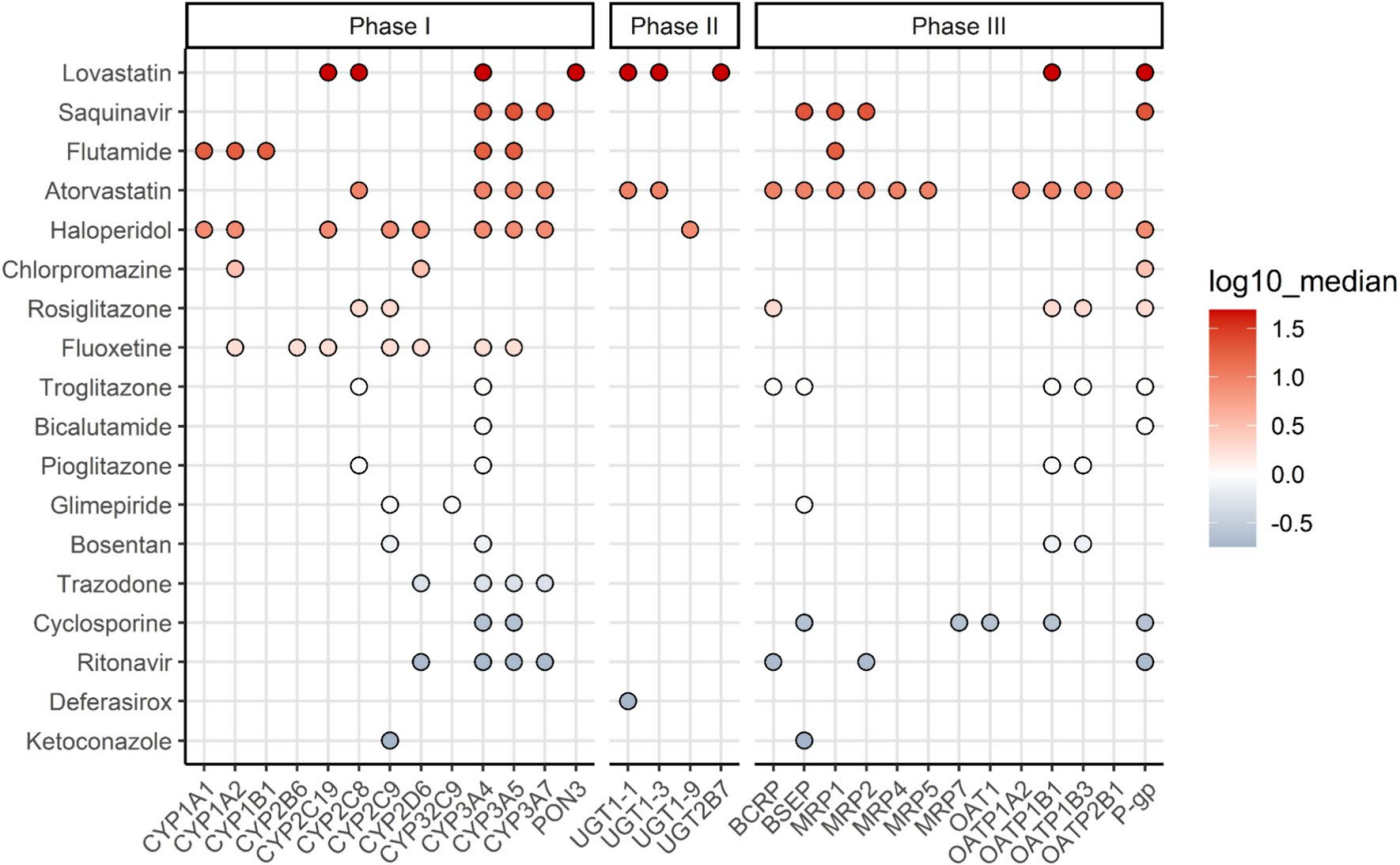
Phase I, II, or III kinetic processes involved in the pharmacokinetics of the 18 selected drugs. To facilitate evaluation of potential parameters that may contribute to deviations in the predicted versus observed *C*_max_, the color of the bullets indicates for the respective drug the logarithm of the median ratio predicted:observed *C*_max_. For the combination of input parameters used for the PBK predictions, see Supplementary Table S3

To facilitate the evaluation of potential processes that may contribute to deviations in the predicted versus observed *C*_max_, an overview of the involved phase I, II, or III processes in the kinetics of the 18 drugs was created (Fig. 4). The color of the bullets indicates the ratio predicted:observed *C*_max_. Interestingly, the *C*_max_ values for the statins lovastatin and atorvastatin were > fivefold overpredicted. These drugs, along with deferasirox and haloperidol, undergo phase II metabolism by several UDP-glucoronosyltransferase (UGT) enzymes. Haloperidol is also overpredicted, but deferasirox is underpredicted. Besides the UGT enzymes, the cytochrome P450 (CYP) enzymes CYP2C8 and CYP3A4 are involved in the metabolism of lovastatin and atorvastatin. Hepatic uptake of lovastatin and atorvastatin occurs through the organic anion transporter (OATP) 1B1. The highest overprediction was observed for lovastatin. All together the overview reveals that the over- or underprediction of the *C*_max_ cannot be ascribed to a specific metabolic phase or enzyme since for all drugs, including the drugs with the highest level of deviation but also the drugs for which accurate predictions were obtained similar phase I, II, and II metabolism and respective isoenzymes seem to be involved.

### Drug PBK model-based predictions of free hepatic concentrations at therapeutic dose levels and their comparison to the *K*_i_ for inhibition of bile acid efflux

Upon evaluation of the drug PBK model predictions for plasma *C*_max_ values, the PBK models were used to predict maximal free hepatic concentrations of the drugs (assumed to be equal to free concentrations of the drugs in venous blood leaving the liver) at therapeutic dose levels. In Table 2, these predicted maximal free hepatic concentrations of the drugs are compared to the respective *K*_i_ values for drug-mediated inhibition of hepatic bile acid efflux by presenting the ratio between these two values. From these ratios, it follows that not all drugs when dosed at their therapeutic level will result in maximal free hepatic concentrations able to reach the *K*_i_ (ratio equals at least 1) and thus will not result in effective inhibition of bile acid efflux. The PBK models can also be used to predict the dose levels required to reach maximal free hepatic concentrations of the drugs that reach the *K*_i_, and these dose levels are also presented in Table 2. Comparison of these dose levels to the therapeutic dose levels corroborates that for some drugs, therapeutic dose levels are high enough to induce maximal free hepatic concentrations in the range or above the *K*_i_, resulting in inhibition of bile acid efflux, while for others higher than therapeutic dose levels would be needed. For cyclosporine for example, the ratio between the maximal free hepatic concentrations and the *K*_i_ amounts to 12.9 and the therapeutic dose level is 11-fold higher than what would be needed to reach the *K*_i_ while for others, like bosentan, this ratio amounts to 0.03, indicating that a 34-fold higher than therapeutic dose level would be needed to reach the *K*_i_.

**Table 2:** Therapeutic dose, inhibitory constant (*K*_i_) for inhibition of hepatic bile acid efflux, ratio between the drug PBK model predicted maximal free hepatic concentration at therapeutic dose level (assumed to be equal to free concentration in venous blood leaving the liver) and the *K*_i_, ratio between the drug PBK model predicted area under the curve (AUC) free hepatic concentration at therapeutic dose level, the predicted dose required to obtain a maximal free hepatic concentration of the drug equal to *K*_i_ and the AUC above the *K*_i_

Drug	Therapeutic dose (mg/kg body weight)^a^	*K*_i_ (nM)^b^	Ratio internal maximal liver concentration: *K*_i_	Ratio internal liver AUC: *K*_i_ (h)	Dose required to reach *K*_i_ (mg/kg body weight)	AUC above the *K*_i_ (µmol/L h)
Atorvastatin	1.143	112	0.15	1.26	8.0	0.00
Bicalutamide	0.714	979	0.22	1.85	3.0	0.00
Bosentan	3.571	493	0.03	0.39	123	0.00
Chlorpromazine	28.57	460	2.56	2.20	11	0.36
Cyclosporine	15.00	4.88	12.9	253	1.2	1.12
Deferasirox	20.00	505	505	0.51	0.19	103
Fluoxetine	0.635	320	0.80	2.29	0.8	0.00
Glimepiride	0.086	603	0.03	0.31	2.6	0.00
Haloperidol	0.214	1134	0.11	0.28	2.0	0.00
Ketoconazole	17.14	173	5.16	26.0	3.5	1.26
Pioglitazone	0.643	631	0.91	14.8	0.7	0.00
Ritonavir	17.14	28.0	4.88	38.3	3.5	0.44
Rosiglitazone	0.114	8.18	0.47	10.8	0.3	0.00
Trazodone	8.571	1919	1.28	9.88	6.5	0.20
Troglitazone	11.43	64.0	2.20	12.4	5.0	0.12

^a^Therapeutic dose is the maximal prescribed daily dose and obtained from the supplier’s information

^b^*K*_i_ is the inhibitory constant of bile acid efflux inhibition induced by the drugs. The *K*_i_ was obtained by measuring glycocholic acid efflux in an assay using primary hepatocytes in suspension (Zhang et al. 2016)

These results already explain why defining the *K*_i_ (or IC_50_) for bile acid efflux inhibition defines the hazard but does not predict the risk on developing cholestasis. However, it is also important to consider that not only a comparison of the maximal free hepatic concentrations to the respective *K*_i_ values is relevant to obtain insight in chances on effects on bile acid efflux and accumulation, but also the duration of this situation where free hepatic concentrations are in the range of the *K*_i_ or above.

**Fig. 5:**
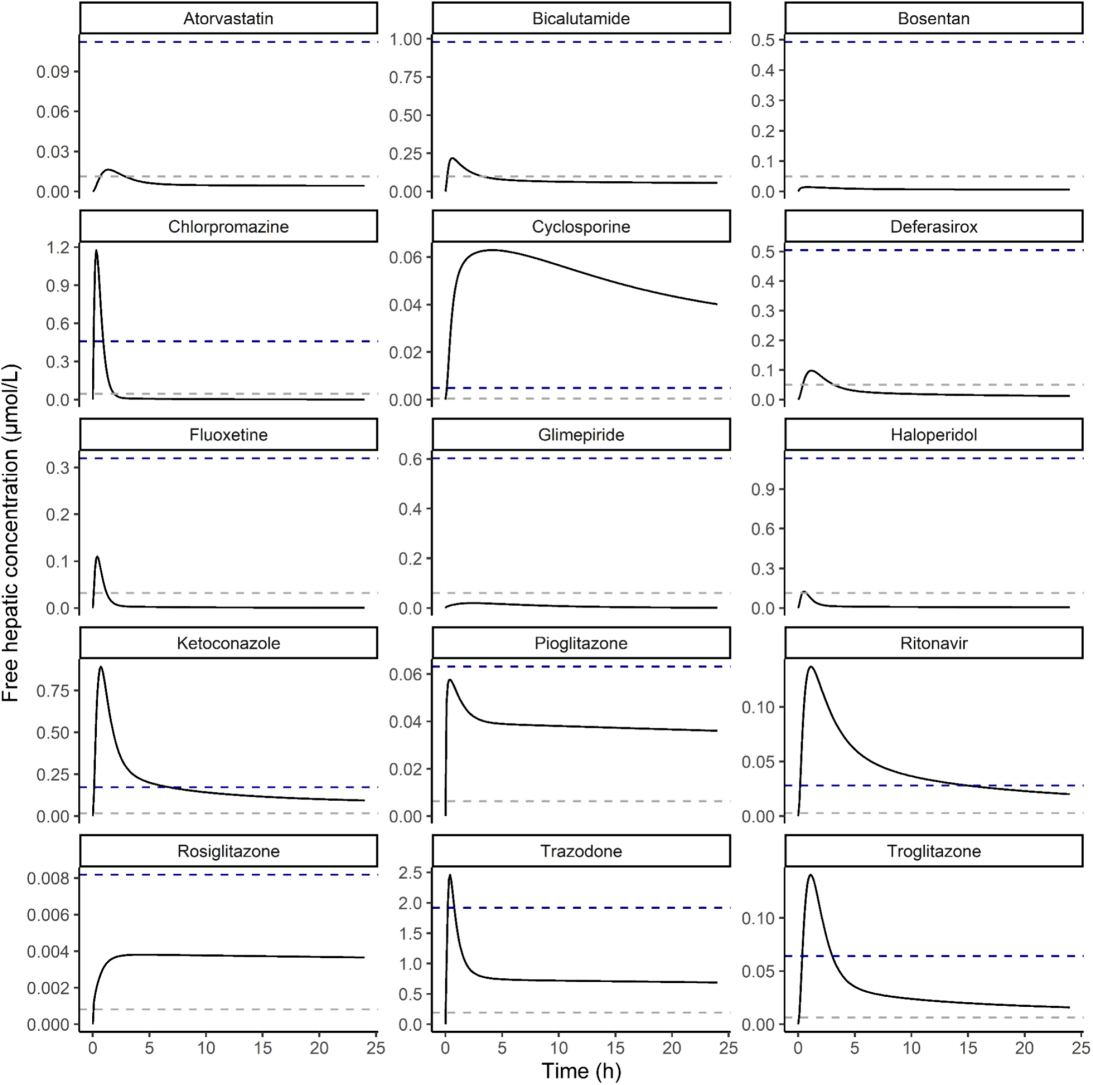
Free hepatic drug concentration over time at therapeutic dose level. Blue dashed line = *K*_i_, gray dashed line = 10% of *K*_i_

To further study these aspects, Fig. 5 presents the drug PBK model-based predictions for the free hepatic drug concentration over time at therapeutic dose levels including a comparison to the respective *K*_i_ values. From these results, it follows that for some drugs, the therapeutic dose will result in free hepatic drug concentrations over the whole 24 h time frame (far) below the respective *K*_i_ in some cases not even reaching 10% of the *K*_i_, the latter being a concentration at which for a competitive inhibitor less than 10% inhibition can be expected. The curves also reveal that for drugs for which at the therapeutic dose level, the maximum free hepatic drug concentration will be reached or even exceed the *K*_i_, the overall time frame during which concentrations in the range or above the *K*_i_ can be expected will depend on the rate of clearance. Comparison of the data for chlorpromazine and troglitazone, for example, reveals that, although the ratio between the predicted maximum free hepatic concentration and the *K*_i_ is comparable for both drugs, amounting to 2.56 and 2.20 respectively, the time frame during which for troglitazone the free hepatic concentrations are in the range of the *K*_i_ is substantially larger than for chlorpromazine, due to the far more efficient clearance of chlorpromazine. This indicates that for prediction of the risk of cholestasis not the ratio between the maximum free hepatic concentration of the drug at therapeutic dose level and the *K*_i_, but rather the ratio between the AUC of the drug compared to the *K*_i_ is of importance. Therefore, Table 2 also presents the AUC/*K*_i_ ratios at therapeutic dose levels for the different drugs.

### PBK model predictions for drug-induced bile acid accumulation

**Fig. 6:**
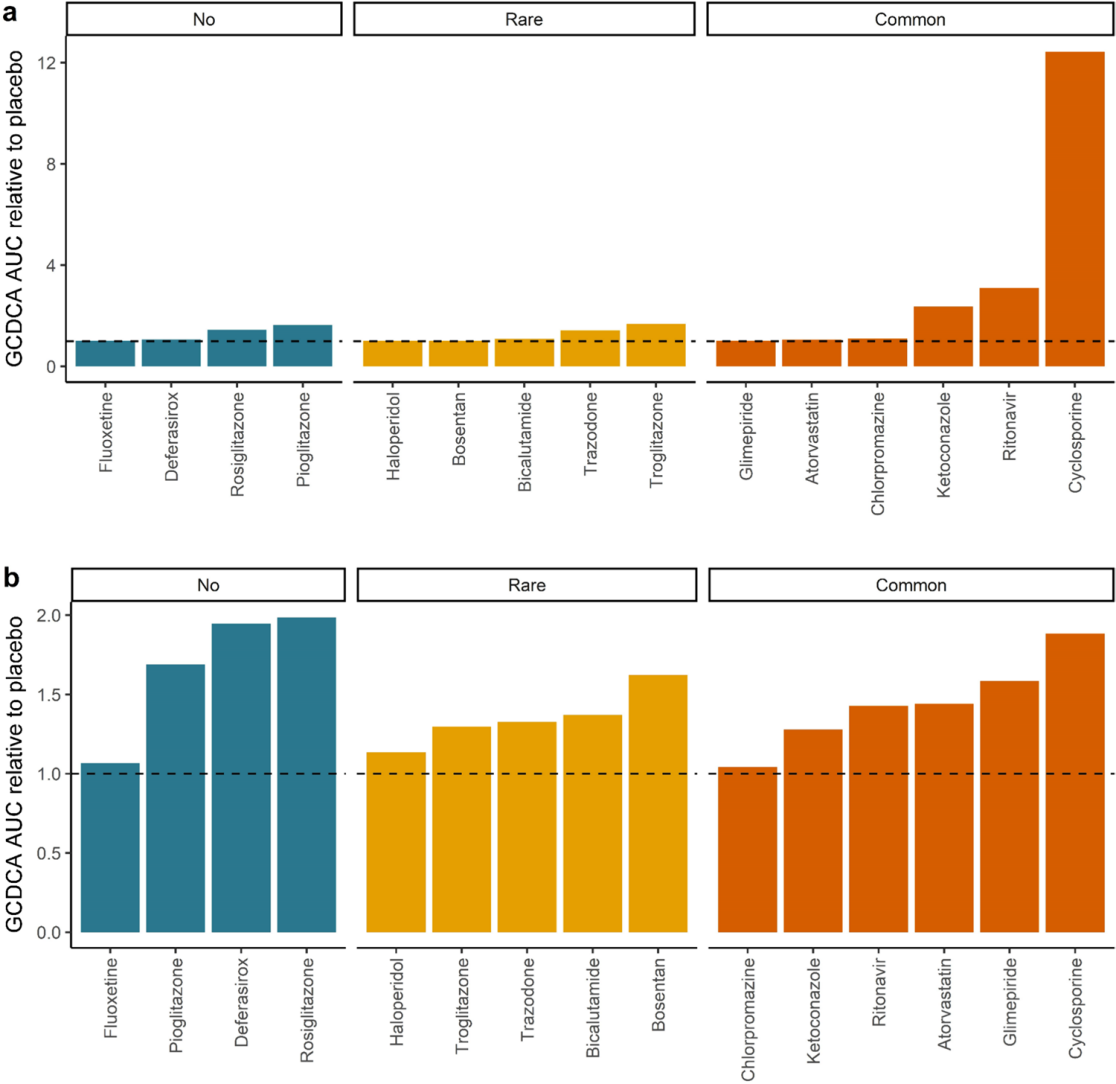
PBK model predicted hepatic glycochenodeoxycholic acid (GCDCA) accumulation at **a** the maximal therapeutic dose level or **b** the dose at which the maximum free hepatic concentration reaches the *K*_i_ (see Table 2). The dashed line indicates the placebo (set to 1). *AUC* area under the curve

**Fig. 7:**
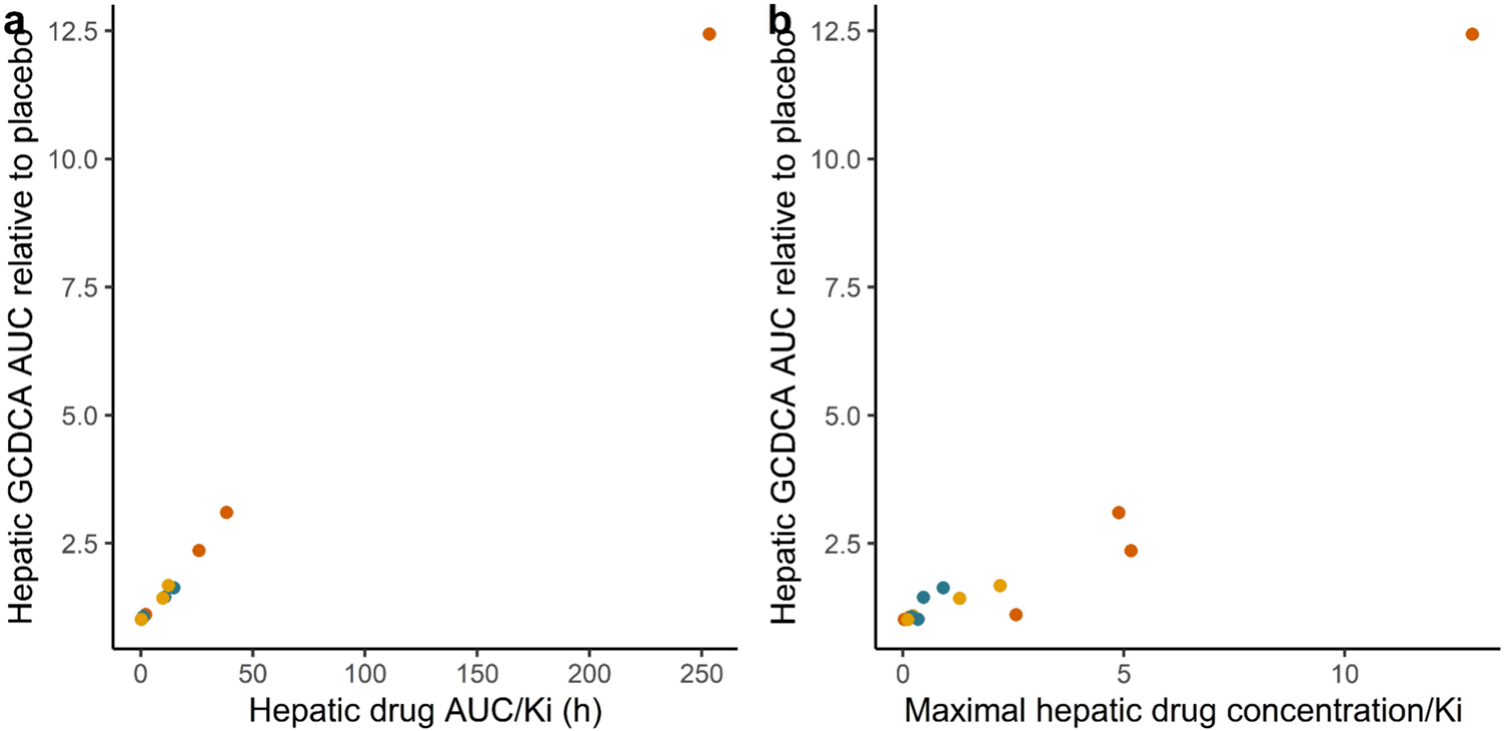
Hepatic glycochenodeoxycholic acid (GCDCA) area under the curve (AUC) relative to placebo versus **a** the hepatic drug concentration AUC/*K*_i_ and **b** maximal hepatic drug concentration/*K*_i_. Simulations were done at therapeutic dose levels

To provide insight in the consequences of the drug kinetic profiles for bile acid accumulation, PBK model predictions for drug-induced bile acid accumulation were made by linking the drug PBK models to a PBK model for bile acid kinetics. Figure 6 presents the predicted GCDCA accumulation at both the therapeutic dose levels as well as at the dose levels at which the maximum free hepatic concentration was predicted to reach the *K*_i_ (Table 2). These results confirm that for several of the drugs, therapeutic dose levels would not result in substantial bile acid accumulation in line with the incidence report for cholestasis being not or rarely reported. When dose levels would be at dose levels where the free maximum hepatic concentrations would be at the *K*_i_, bile acid accumulation would be always less than twofold compared to the placebo and in some cases not observed at all. Of interest to note is also that only for some of the drugs for which cholestasis is observed commonly, especially cyclosporine, ritonavir, and ketoconazole, bile acid accumulation at therapeutic dose levels is higher than what is predicted for the placebo (Fig. 6a). Comparison to the bile acid accumulation predicted for these drugs at dose levels where the *K*_i_ is reached (Fig. 6b) reveals that this higher bile acid accumulation at therapeutic dose levels can be ascribed to the fact that the therapeutic dose level is higher than the dose levels where the *K*_i_ is reached (Table 2). The results obtained also reflect that this PBK model-based prediction accounts for the effects of the drug on the bile acid efflux during the whole 24 h interval thus also taking into account differences in drug clearance. This is again illustrated by the differences between chlorpromazine and troglitazone for which the ratio between the predicted maximum free hepatic concentration at therapeutic dose level and the *K*_i_ is comparable (Table 2). At both simulated dose levels, chlorpromazine, in spite of its ability to inhibit bile acid efflux, was predicted to not result in hepatic GCDCA accumulation, while troglitazone, with less efficient clearance and a higher AUC, was predicted to result in increased hepatic bile acid levels (Fig. 6). To further illustrate the importance of the size of the AUC as compared to the *K*_i_ for predicting whether bile acid accumulation is to be expected, Fig. 7a presents the PBK model predicted GCDCA accumulation (expressed as fold change in the AUC of GCDCA compared to the placebo) versus the ratio of the AUC of the drug versus the *K*_i_, showing that the PBK modeling indeed adequately accounts for the effect of the varying concentration of the drug on bile acid accumulation overtime, while when using the ratio of the maximum free hepatic concentration of the drug and the *K*_i_ as the descriptive parameter no adequate prediction is obtained (Fig. 7b).

Finally, it is also of interest to note that the PBK model-based predictions for bile acid homeostasis presented in Fig. 6 do not in all cases reflect the frequency of occurrence of cholestasis at therapeutic doses of the drug. While for some compounds, it is clear that therapeutic dose levels are high enough to cause bile acid accumulation; for others, the therapeutic dose levels are too low to induce this effect. There are also drugs for which bile acid accumulation is predicted to occur, while there is no or only rare reported incidence of cholestasis, while for some drugs for which occurrence of cholestasis is common bile acid accumulation at therapeutic dose level is limited or even absent. Of special interest is the apparent difference between the three thiazolidines troglitazone, rosiglitazone, and pioglitazone. The kinetic profiles predicted for these compounds by the generic PBK model show substantial differences and especially a lack of effective clearance at prolonged time intervals for rosiglitazone and pioglitazone, resulting in potentially unrealistically high AUC levels for these drugs by the generic PBK model.

This suggests that using the AUC above the *K*_i_ as a measure to predict the occurrence of cholestasis may be a better approach. Table 2 lists these predicted AUC above *K*_i_ values for the various drugs. This would predict only the following drugs of the series of 15 drugs studied to induce cholestasis at their therapeutic dose in the order: ketoconazole > cyclosporine > > ritonavir > chlorpromazine > trazodone > troglitazone. It is of interest to note that these first 4 compounds are all listed as commonly inducing cholestasis, while trazodone and troglitazone rarely induce cholestasis. This would suggest that the AUC above the *K*_i_ might be the best parameter to predict the risk of cholestasis. The fact that this AUC above the *K*_i_ would not identify the common occurrence of cholestasis for atorvastatin and glimepiride might be related to the fact that another mode of action than inhibition of bile acid efflux is underlying the effect, since at therapeutic dose levels, these two drugs were predicted to never reach free hepatic concentrations that would cause efficient inhibition of bile acid efflux.

### Increased bile acid pool size and reduced BSEP abundance are potential synergistic risk factors for cholestasis

**Fig. 8:**
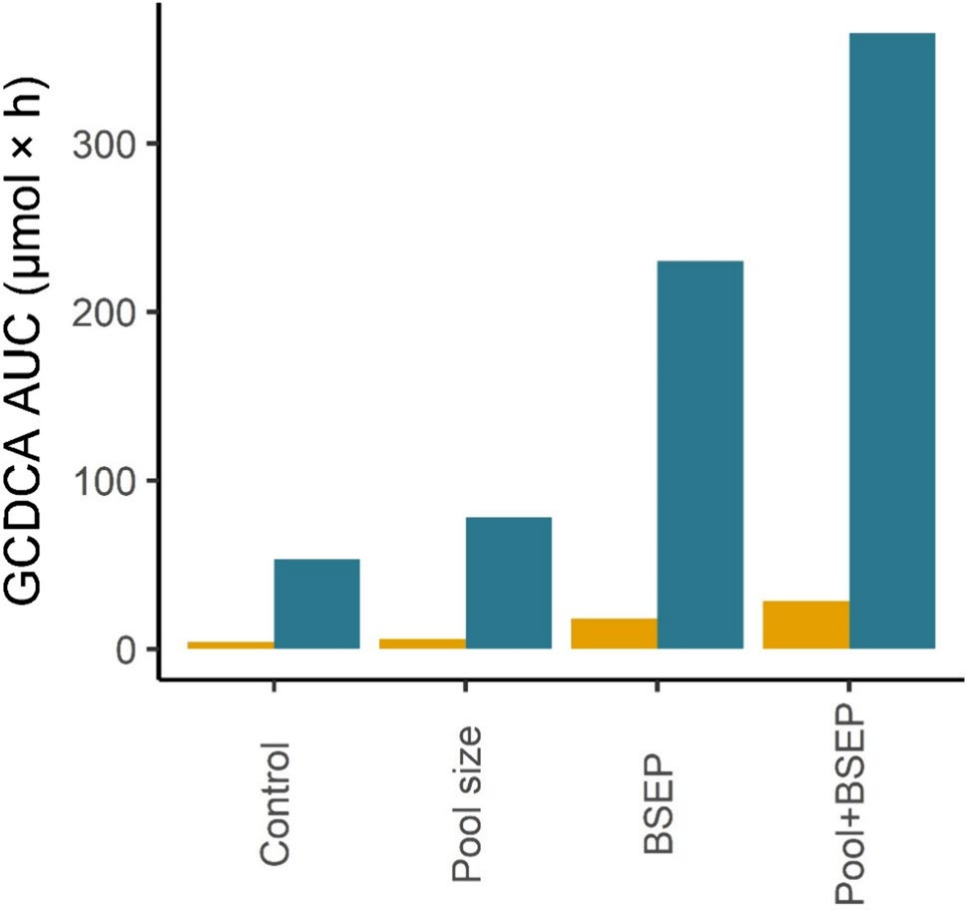
Area under the curve (AUC) of GCDCA levels in liver intracellular water upon administration of the maximal prescribed daily dose of cyclosporine in different sensitive individuals. Yellow = placebo, blue = cyclosporine. Control = reference individual, pool size = 1.5-fold increased GCDCA pool size compared to the reference, BSEP = low BSEP abundance, Pool + BSEP = individual with a 1.5-fold increased GCDCA pool low BSEP abundance. *GCDCA* glycochenodeoxycholic acid

The PBK modeling of drug-induced bile acid accumulation presented so far did not yet take into account factors that may cause individuals to become sensitive toward bile acid accumulation. In subsequent PBK modeling studies, it was investigated to what extent an augmented bile acid pool size and low BSEP abundance are potential risk factors for the development of cholestasis upon exposure to selected drugs. To this end, first the effects of cyclosporine administration for individuals with an increased total bile acid pool or decreased BSEP levels or both were simulated (Fig. 8). Cyclosporine was selected for these studies, because the free intrahepatic levels of this drug were shown to meet the *K*_i_ threshold upon therapeutic dose levels. For comparison, also the effects of these interindividual modifications on the hepatic GCDCA levels upon placebo treatment were calculated and are presented in Fig. 8 as well. The combination of both an increased pool size and low BSEP abundance resulted in intrahepatic GCDCA levels surpassing the cumulative effects of each factor in isolation. These observations suggest a potential synergistic impact for individuals in which both risk factors are present simultaneously.

### Sensitivity analysis

**Fig. 9:**
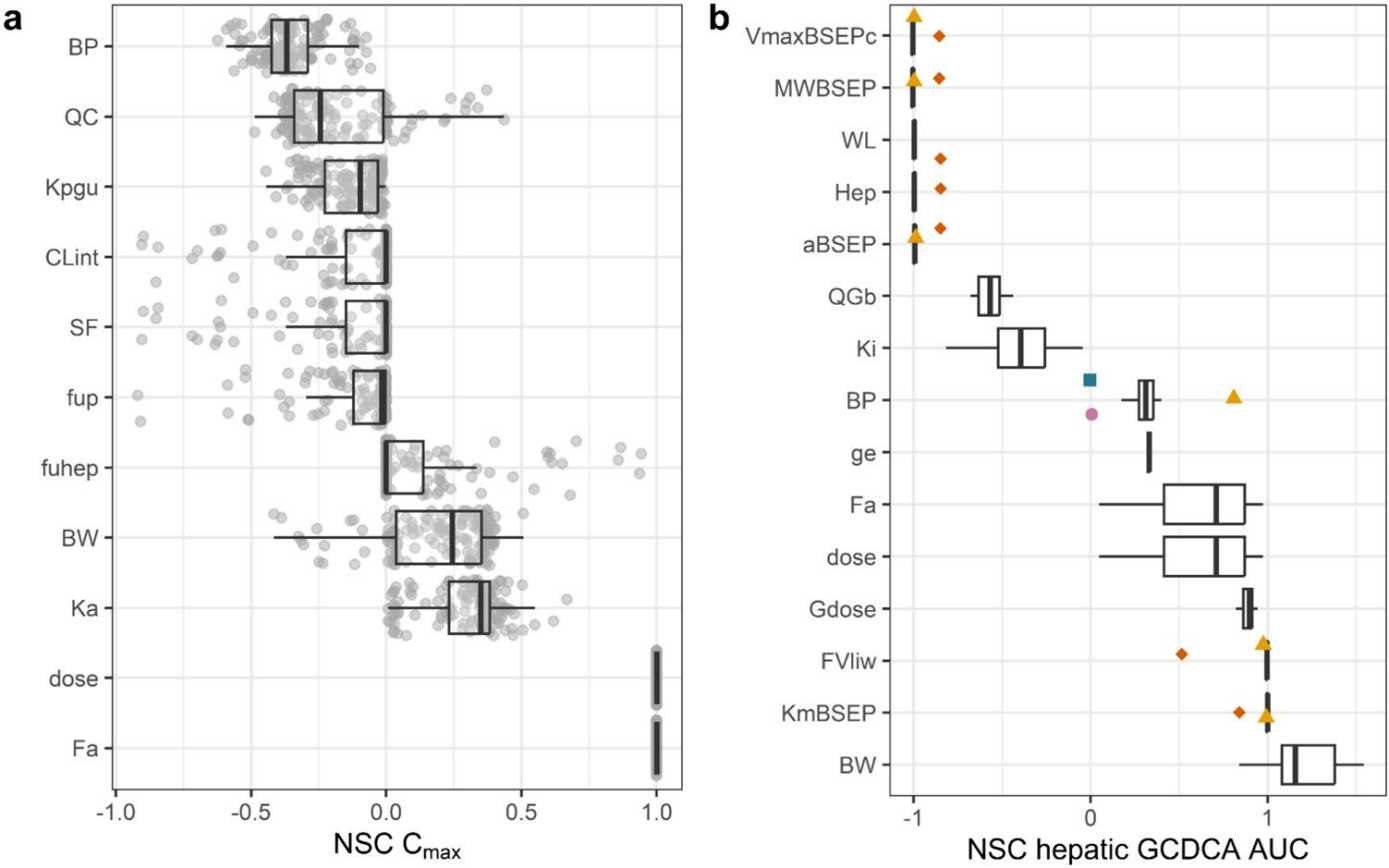
Sensitivity analysis for **a** plasma *C*_max_ of 18 drugs parameterized using different combinations of input parameters and **b** hepatic glycochenodoxycholic acid (GCDCA) accumulation as indicated by the area under the curve (AUC) of hepatic GCDCA after administration of 15 selected reference drugs. Outliers are colored and shaped. Parameters are included when in **a** 15 or more and in **b** 10 or more normalized sensitivity coefficients (NSCs) were < − 0.25 or > 0.25. BP: blood:plasma ratio; QC: cardiac output; Kpgu: plasma:gut partition coefficient; Clint: clearance; SF: scaling factor for clearance; fup: fraction unbound in plasma, fuhep: fraction unbound to hepatocytes; BW: body weight; Ka: absorption rate constant of the drug; dose: dose; Fa: fraction absorbed; *V*_max_BSEPc: maximal rate of BSEP-mediated hepatic GCDCA efflux; MWBSEP: molecular weight of BSEP; WL: weight of liver; hep: hepatocellularity; aBSEP: BSEP abundance; Qgb: fraction of GCDCA going directly to the gallbladder; *K*_i_: inhibitory constant of hepatic bile acid efflux; ge: gallbladder ejection rate; Gdose: amount of GCDCA in gallbladder at *t* = 0; FVliw: fraction of intracellular water in liver; *K*_m_BSEP: Michaelis–Menten constant of BSEP-mediated hepatic GCDCA efflux. square = chlorpromazine, circle = fluoxetine, up-pointing triangle = pioglitazone, diamond suit = rosiglitazone

The sensitivity analysis (Fig. 9a) revealed that especially the fraction absorbed (Fa) and dose of the drug have strong positive influence on the plasma *C*_max_ of the drugs, and that the blood:plasma ratio (BP) has a strong negative influence. Cardiac output (QC) and body weight (BW) could have a positive or negative influence on the plasma *C*_max_ of the drugs, depending on which drug was simulated and the parametrization of the PBK model. Figure 9b reveals that the parameters related to the bile acid homeostasis typically had a stronger influence on the hepatic GCDCA AUC than drug-specific parameters. The most influential drug-specific parameters were *K*_i_ for hepatic efflux inhibition (*K*_i_), fraction absorbed (Fa), and dose of the drug. Parameters related to the maximal rate of BSEP-mediated hepatic GCDCA efflux or its scaling had a strong influence on the hepatic GCDCA AUC. A boxplot was generated of the normalized sensitivity coefficients for all 15 drugs. Some NSC values were ± 1.5 × the interquartile range and thus considered an outlier. Normalized sensitivity coefficients for rosiglitazone were most often considered outliers followed by pioglitazone.

## Discussion

Accurate predictions of internal dosimetry are of paramount importance in driving the acceptance of advanced (animal-free) testing methodologies for chemical safety evaluations. Internal dosimetry predictions are also instrumental in bridging the gap between in vitro toxicity and in vivo dose–response relationships or drug potency data. The present study aims at development of a new approach methodology (NAM) to predict drug-induced cholestasis, as a result of hepatic efflux inhibition and subsequent bile acid accumulation. To this end, a generic PBK model was built to predict hepatic concentrations of 18 selected drugs. For 15 of these drugs, the predicted *C*_max_ was within tenfold of the observed *C*_max._ The predicted internal hepatic dose level of these 15 drugs was incorporated in a bile acid PBK model describing the synthesis, circulation, and excretion of the exemplary bile acid GCDCA. The intrahepatic GCDCA accumulation was determined as a measure for cholestatic potency.

The generic PBK models were parameterized using different in vitro and in silico input parameters and the plasma *C*_max_ predictions were validated with in vivo plasma *C*_max_. Different methods were applied to define the parameters required to model the drug kinetics by the generic PBK model. Tissue-partitioning was predicted using the quantitative-property-property relationships as described by Berezhkovskiy (2004) or Rodgers and Rowland (2006). For acidic drugs, i.e., pKa < 6, the method used for prediction of the tissue:partition coefficients had a major effect on predicted plasma *C*_max_. For 2 out of 4 strongly acidic drug in our dataset, the predictions were closest to the in vivo data when the method of Rodgers and Rowlands was used, while for the remaining 2 strongly acidic drugs, the closest fit was achieved with Berezhkovksiy’s method. These observations suggest that the most predictive approach to simulate plasma *C*_max_ cannot be determined by a drug’s physicochemical properties alone but that model parameterization should be evaluated on a case-by-case basis, and include drug-specific processes where necessary to enhance predictive accuracy.

In vivo clearance predictions were based on studies with primary human hepatocytes (PHHs) and the in silico tool pkCSM. Interestingly, pkCSM resulted in *C*_max_ predictions similar to the predictions made with in vitro clearance data. If any differences were observed between the two different inputs, the pkCSM tool resulted in higher, i.e., more conservative, predictions than the in vitro clearance. These findings provide support for the use of the pkCSM tool also for pioglitazone, rosiglitazone, and deferasirox for which no in vitro clearance data obtained from PHHs were available. PHHs retain all phase I and phase II metabolic enzymes and co-factors necessary for metabolic clearance and transporter function and are considered the golden standard for hepatic clearance predictions (Richert et al. 2006). Comparison of the PBK model predictions of the present study based on the clearance data obtained with the various PHH models indicates adequate predictions and confirms the validity of the in vitro model used to quantify drug clearance. Nevertheless, in vitro clearance predictions typically underestimate in vivo clearance for highly bound chemicals, even after correcting for the fraction unbound in plasma (Jones et al. 2022; Hallifax et al. 2010). An underestimation of the clearance is unlikely to have affected the risk prioritization in the current work. The current study included only three drugs that were > 90% bound to plasma, i.e., fluoxetine, cyclosporine, and haloperidol. Here, the use of PHHs may have resulted in an underestimated CL_int_ and thus higher and more conservative predictions of intrahepatic drug levels. Despite being potentially conservative, the maximal free hepatic concentration of fluoxetine and haloperidol did not exceed the *K*_i_. Consequently, we predicted low risk upon therapeutic fluoxetine or haloperidol dosing. Finally, the cyclosporine PBK model was parameterized based on pkCSM, so these results were not affected by a potential artifact in the in vitro clearance. Further research is needed to determine the most suitable way to predict clearance of highly bound compounds. Potentially, in silico tools may provide accurate and quick clearance predictions for highly bound compounds, especially when trained based on human in vivo clearance data with a well-defined chemical applicability domain. The use of empirical scalars to correct for known and unknown differences between the in vitro and in vivo situation has been shown a promising alternative to predict in vivo hepatic clearance (Jones et al. 2022).

The complete set of evaluated drugs consisted of 18 drugs. For 13 drugs, the predictions made by the generic PBK model were within a fivefold range, and for 7 drugs, the predictions were within a twofold range of the corresponding in vivo data. Typically, regulatory contexts demand predictions by a drug-specific PBK model within a twofold range of the in vivo data (Peters and Dolgos 2019); however, given the large variability within reported human in vivo kinetic data, this requirement might be excessively rigorous. Thus, deviations may in part be due to the variability within reported human in vivo biokinetic data. For saquinavir, for example, in vivo *C*_max_ values ranging from 5.4 to 66.1 µg/L were observed at similar dose levels (Frohlich et al. 2004; Vella and Floridia 1998) indicating variability in the available human in vivo data as a potential reason for the relatively large fold differences between predicted and reported *C*_max_ values. Biological, technical and analytical interstudy differences could contribute to this variability, underpinning the need for harmonized in vivo biokinetic study protocols and the need to understand at least the main drivers of interindividual differences. Only 3 drugs (i.e., lovastatin, saquinavir, and flutamide) were excluded from further analysis toward potential bile acid accumulation, because the generic PBK model overpredicted their plasma *C*_max_ by > tenfold. Aside from the large differences in human in vivo data contributing to the discrepancies, the generic, minimal structure of the PBK model is unlikely to make precise predictions. Generic PBK models describe the kinetic aspects for all drugs in a similar way with a limited amount of input parameters. This method is less time-consuming than developing a chemical-specific PBK model which often requires experimental determination of specific kinetic parameters for, e.g., transporter-mediated renal excretion. Even though less-precise, predictions within tenfold may still be considered relevant (Punt et al. 2021). When higher confidence is needed, chemical-specific, or perhaps class-specific PBK models need to be developed. For example, saquinavir and flutamide are multidrug resistance protein (MRP)-1 substrates and it can be speculated that by incorporating MRP-1 mediated renal and/or biliary clearance in the PBK model, the predictions would be improved. Lovastatin *C*_max_ is probably overestimated, because extrahepatic clearance is not (sufficiently) considered, resulting in an underprediction of total clearance. No in vitro lovastatin intrinsic clearance was measured using PHHs (Pearce et al. 2017), while the total in vivo lovastatin clearance was reported to be 451 L/h (Zhou et al. 1995). pkCSM predicted a higher total clearance than was measured using PHHs, but the predicted clearance only amounted to 36 L/kg after extrapolation to the in vivo situation. pkCSM might not be suitable to accurately predict total clearance of drugs with substantial extrahepatic clearance. Lovastatin is metabolized through glucuronidation, lactonization, and cytochrome P450-mediated oxidation which can take place outside the liver (Reig-Lopez et al. 2021). Glucuronidation is catalyzed by UDP-glucuronosyltransferases (UGTs) and these are widely expressed in various tissues, including liver, kidney, lung, and intestine (Tukey and Strassburg 2000; Naritomi et al. 2015). Lactonization can occur as a spontaneous process at pH < 6 or mediated by plasmatic paraoxonase (PON) at pH > 6 (Reig-Lopez et al. 2021). Underestimation of the clearance provides a conservative estimate of the *C*_max_.

Upon gaining sufficient confidence in the PBK predictions for 15 out of 18 drugs, we employed the PBK models to predict free intrahepatic drug concentrations and incorporated these into the bile acid PBK model to predict the effects of the drugs on hepatic bile acid accumulation resulting from inhibition of bile acid efflux. Hepatic accumulation at therapeutic dose levels was simulated for the commonly cholestatic drugs cyclosporine, ritonavir, and ketoconazole. Interestingly, no hepatic GCDCA accumulation was predicted at therapeutic dose levels of atorvastatin, chlorpromazine, and glimepiride, even though these drugs were classified as common causes of cholestasis. It is hypothesized that these drugs cause cholestasis through mechanism(s) that cannot be fully captured by the short-term (1 h) assay with a suspension-cultured PHH assay. The PHH assay is most suitable to study short-term competitive transporter inhibition. According to the cholestasis AOP, the mechanism(s) or molecular initiating events (MIEs) could involve transporter, hepatocellular, and bile canalicular changes (Vinken et al. 2013; Gijbels et al. 2020). Chlorpromazine probably induced cholestasis through hepatocellular changes that can be observed only after several hours of incubation. It was shown that HepaRG cells exposed to 50 µM of chlorpromazine for 4 h lost ~ 50% of hepatocellular tight junctions (Morgan et al. 2019). Glimepiride induced intrahepatic bile canalicular dilatation in a human clinical case study (Omar et al. 2009), which can impossibly be captured in a system with suspension PHHs. Atorvastatin-induced cholestasis is not fully understood, but it is has been speculated that immune-allergic reactions or ROS formation are involved (Karahalil et al. 2017). Furthermore, the different DILI patterns (hepatocellular/cholestasis/mixed) reported after atorvastatin treatment suggest that its mode of action is multifaceted and that a battery of tests is required for an NAM-based risk assessment (Averbukh et al. 2022).

In our previous study, we observed a 60% increase in the liver GCDCA upon bosentan treatment, while in the current work, no substantial increase was observed (de Bruijn et al. 2022). The discrepancy between the two studies can be attributed to variations in dosage and tissue partitioning, ultimately leading to a lower free intrahepatic bosentan concentration. In our earlier study, a clinical trial-like dosage of 500 mg twice a day was simulated (Fattinger et al. 2001), while in the current study, the maximal prescribed daily dose of 250 mg once a day was used. Furthermore, in our previous investigation, an experimental log*P* value of 3.1 was employed (Meyer 1996), while in the current study, a log*P* value of 5.5, predicted using Chemaxon, was used. This change, combined with a slightly higher pKa value (5.5 versus 5.8), led to a tenfold increase in the liver:plasma partition coefficient, as calculated using the method of Rodgers and Rowlands. Besides, the in silico calculated fraction unbound decreased by fivefold due to these changes in physicochemical parameters. Taking the influence of the physicochemical parameters and the dose together, this resulted in a decreased free hepatic bosentan concentration in the current manuscript compared to the previous study and thus a reduced effect on hepatic GCDCA levels. These findings stress the importance of accurate estimates of lipophilicity.

Inhibition of hepatic bile acid efflux is a hazard for cholestasis risk, but the results of the present study clearly demonstrated that predicting the risk cannot be based on the IC_50_ or *K*_i_ for inhibition of hepatic bile acid efflux alone. PBK modeling of the intracellular hepatic drug concentration time profile and its comparison to the *K*_i_ appeared to be the best way to predict the cholestatic potential with especially the AUC above the *K*_i_ providing a better prediction than the total AUC/*K*_i_ ratio or the *K*_i_ or IC_50_ as such. This indicates that one has to take into account not only the drug’s potency to inhibit the bile acid efflux, but also the external dose level and its kinetics. In addition, the individual’s susceptibilities were shown to influence the risk with people with a higher bile acid pool size and low BSEP abundance being more susceptible. The developed combined drug and bile acid PBK models incorporate all this information and predict drug-induced cholestasis as a result of hepatic transporter inhibition. For a complete risk assessment of cholestasis, also MIEs focusing on hepatocellular and bile canalicular changes need to be included. Future research should focus on validation and standardization of these assays and quantitatively coupling the measured MIEs to cholestasis risk. The current results provide a proof-of-principle of a PBK model to bridge the gap between in vitro potency to inhibit hepatic bile acid efflux and in vivo cholestasis risk prioritization.

### Supplementary Information

Below is the link to the electronic supplementary material.

Supplementary file 1 (DOCX 742 KB)

## Data Availability

The datasets generated during and/or analysed during the current study are available in the Github repository. https://github.com/Veronique-de-Bruijn/PBK-model-cholestasis.git

## Abbreviations

ADR: Adverse drug reaction
AO: Adverse outcome
AOP: Adverse outcome pathway
AUC: Area under the curve
BSEP: Bile salt export pump
CL_int_: Intrinsic clearance
C_max_: Maximal concentration in plasma
CYP: Cytochrome P450
DILI: Drug-induced liver injury
FDA: Food and Drug Administration
F_up_: Fraction unbound in plasma
GCDCA: Glycochenodeoxycholic acid
MIE: Molecular Initiating Event
MRP: Multidrug resistance protein
NAM: New approach methodology
OATP: Organic anion transporter
PBK: Physiologically based kinetic
PON: Paraoxonase
QSAR: Quantitative structure–activity relationship
UGT: UDP-glucuronosyltransferases

## Footnotes

1 https://github.com/Veronique-de-Bruijn/PBK-model-cholestasis.git.
