# Supplementary material for: From hazard to risk prioritization: a case study to predict drug-induced cholestasis using physiologically based kinetic modeling
Source: Arch Toxicol. 2024 May 17;98(9):3077–95. doi: 10.1007/s00204-024-03775-6 (PMC11324677; doi:10.1007/s00204-024-03775-6)
Supplement: Supplementary file 1 — (DOCX 742 KB) [file 204_2024_3775_MOESM1_ESM.docx]

# Supplementary material


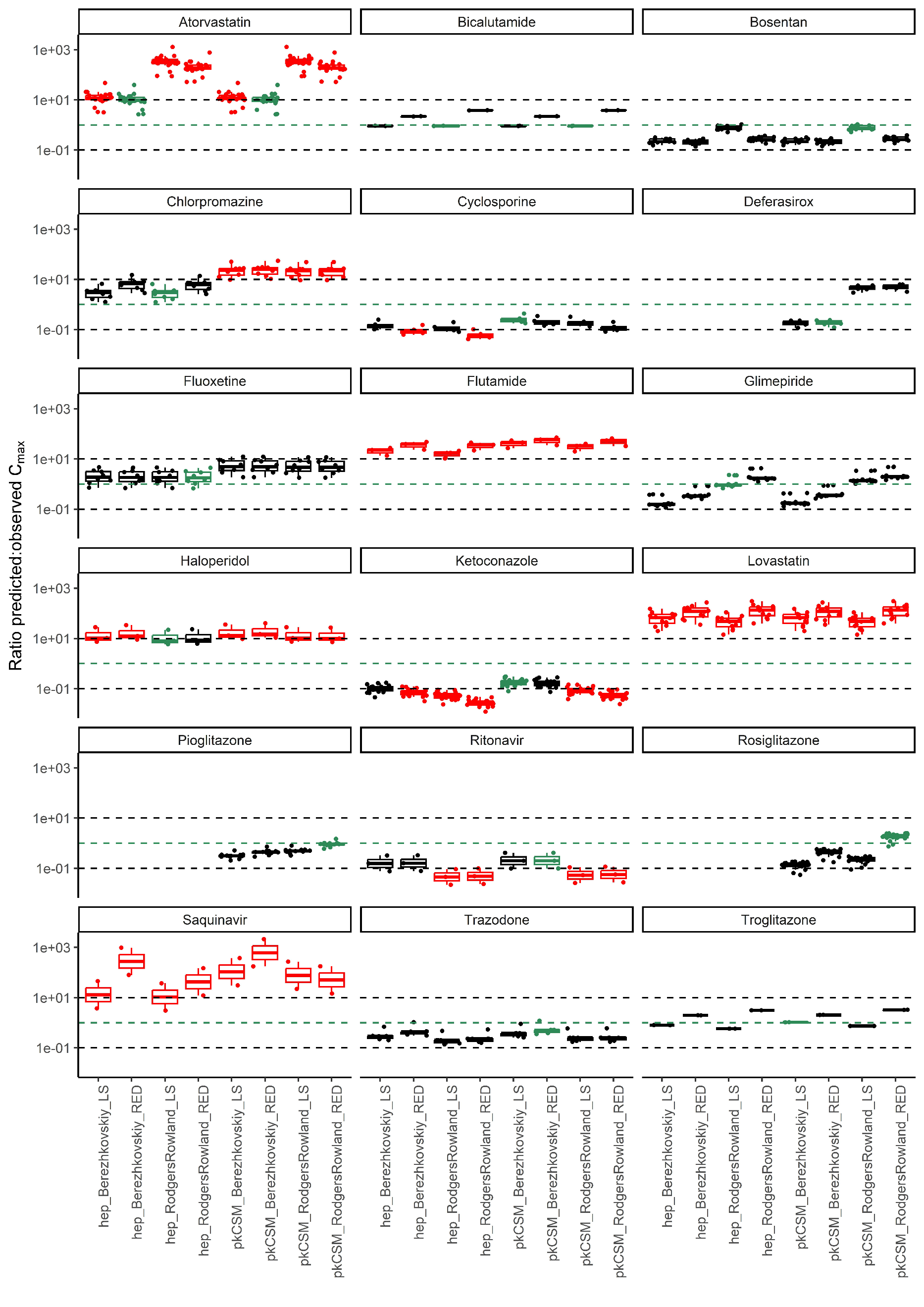


**Figure S1** Ratio predicted:observed C_max_ for the selected drugs for up to 8 different combinations of input parameters. Clearance: hep=primary hepatocytes, pkCSM=in silico clearance, partition coefficients: Berezkovskiy or Rodgers and Rowlands, fraction unbound in plasma: LS=Lobell Sivarajah, RED=rapid equilibrium dialysis. Red: the median is >10 fold over- or underpredicted, black: median is within 10-fold, green: the median with the smallest fold-difference. In case two methods resulted in exactly the same method, the in silico methods were chosen for further simulations.

**Table S1** Physicochemical properties, in vitro and in silico intrinsic clearance (CLint) for the 18 selected drugs

| Drug | pKa1 | pKa2 | logP | *In vitro* CLint (µL/min/10^6^ hepatocytes) | Reference | *In silico* CLint (mL/min/kg body weight) | Reference |
| --- | --- | --- | --- | --- | --- | --- | --- |
| Atorvastatin | 4.31 |  | 5 | 0 | httk | 2.673 | pkCSM |
| Bicalutamide | 11.78 |  | 2.52 | 0 | httk | 3.811 | pkCSM |
| Bosentan | 5.8 | 1.28 | 5.53 | 2.34 | httk | 2.851 | pkCSM |
| Chlorpromazine | 9.2 |  | 4.56 | 146 | (Louisse et al., 2020) | 3.981 | pkCSM |
| Cyclosporine | 11.82 |  | 3.38 | 8.59 | httk | 3.656 | pkCSM |
| Deferasirox | 4.51 | -0.13 | 5.17 |  |  | 3.664 | pkCSM |
| Fluoxetine | 9.4 |  | 4.19 | 36.7 | (Chao et al., 2009) | 4.786 | pkCSM |
| Flutamide | 12.81 |  | 3.4 | 30.4 | httk | 1.146 | pkCSM |
| Glimepiride | 5.62 |  | 2.93 | 3.67 | (Hallifax et al., 2010) | 4.909 | pkCSM |
| Haloperidol | 13.96 | 8.2 | 3.08 | 5.06 | httk | 12.79 | pkCSM |
| Ketoconazole | 6.42 |  | 4.28 | 55 | httk | 3.882 | pkCSM |
| Lovastatin | 14.9 |  | 3.47 | 0 | httk | 8.472 | pkCSM |
| Pioglitazone | 7.32 | 5.63 | 3.1 |  |  | 0.904 | pkCSM |
| Ritonavir | 13.68 | 2.84 | 5.04 | 8.57 | httk | 3.540 | pkCSM |
| Rosiglitazone | 7.37 | 6.4 | 2.86 |  |  | 1.297 | pkCSM |
| Saquinavir | 6.67 | 13.61 | 2.51 | 37.1 | (Wood et al., 2018) | 1.854 | pkCSM |
| Trazodone | 7.49 |  | 3.132 | 7.40 | httk | 4.688 | pkCSM |
| Troglitazone | 7.31 |  | 4.87 | 21.9 | httk | 0.382 | pkCSM |

**Table S2** IC_50_ for bile acid efflux inhibition in primary human hepatocytes (PHH) or BSEP-transfected membrane vesicles (MV).

| Drug | IC_50_ PHH (µM) | Reference | IC_50_ MV (µM) | Reference |
| --- | --- | --- | --- | --- |
| Atorvastatin | 2.6 | Zhang2016 | 13 | (Morgan et al., 2013) |
| Bicalutamide | 22.4 | Zhang2016 | 79.6 | (Morgan et al., 2013) |
| Bosentan | 9.2 | Zhang2016 | 23; 38.1^1^ | (Morgan et al., 2013; Dawson et al., 2012) |
| Cyclosporine | 0.1 | Zhang2016 | 0.5; 0.5^1^ | (Morgan et al., 2013; Dawson et al., 2012) |
| Deferasirox | 11.9 | Zhang2016 | 58.4 | (Morgan et al., 2013) |
| Ketoconazole | 3 | Zhang2016 | 3.4; 2.9^1^ | (Morgan et al., 2013; Dawson et al., 2012) |
| Ritonavir | 0.2 | Zhang2016 | 1.74 | (Morgan et al., 2013) |
| Troglitazone | 0.5 | Zhang2016 | 3; 2.7^1^ | (Morgan et al., 2013; Dawson et al., 2012) |
| Chlorpromazine | 6.4 | Zhang2016 | >100 | (Dawson et al., 2012) |
| Fluoxetine | 5.5 | Zhang2016 | >100 | (Hafey et al., 2020) |
| Glimepiride | 14.1 | Zhang2016 | 15.7 | (Morgan et al., 2013) |
| Haloperidol | 25.3 | Zhang2016 | >100 | (Kock et al., 2014) |
| Pioglitazone | 1.4 | Zhang2016 | 0.3 | (Dawson et al., 2012) |
| Rosiglitazone | 0.2 | Zhang2016 | 2.8;6.4^1^ | (Morgan et al., 2013; Dawson et al., 2012) |
| Trazodone | 43 | Zhang2016 | >100 | (Kostrubsky et al., 2006) |

1 Values from two different literature studies

**Table S3** The combination of input parameters that gives an absolute median ratio predicted:observed C_max_ with the smallest fold difference. >10-fold overpredictions are excluded from further simulations.

| Drug | Clearance | Partition coefficients | Fraction unbound | Median ratio predicted:observed C_max_ |
| --- | --- | --- | --- | --- |
| Atorvastatin | pkCSM | Berezhkovskiy | Equilibrium dialysis | 9.7 |
| Bicalutamide | pkCSM | RodgersRowland | LobellSivarajah | 0.9 |
| Bosentan | pkCSM | RodgersRowland | LobellSivarajah | 0.7 |
| Chlorpromazine | Primary hepatocytes | RodgersRowland | LobellSivarajah | 3.0 |
| Cyclosporine | pkCSM | Berezhkovskiy | LobellSivarajah | 0.2 |
| Deferasirox | pkCSM | Berezhkovskiy | Equilibrium dialysis | 0.2 |
| Fluoxetine | Primary hepatocytes | RodgersRowland | Equilibrium dialysis | 1.8 |
| Glimepiride | Primary hepatocytes | RodgersRowland | LobellSivarajah | 0.9 |
| Haloperidol | Primary hepatocytes | RodgersRowland | LobellSivarajah | 8.2 |
| Ketoconazole | pkCSM | Berezhkovskiy | LobellSivarajah | 0.2 |
| Pioglitazone | pkCSM | RodgersRowland | Equilibrium dialysis | 0.9 |
| Ritonavir | pkCSM | Berezhkovskiy | Equilibrium dialysis | 0.2 |
| Rofecoxib | pkCSM | Berezhkovskiy | LobellSivarajah | 0.6 |
| Rosiglitazone | pkCSM | RodgersRowland | Equilibrium dialysis | 1.9 |
| Trazodone | pkCSM | Berezhkovskiy | Equilibrium dialysis | 0.5 |
| Troglitazone | pkCSM | Berezhkovskiy | LobellSivarajah | 1.0 |
| >10-fold overpredicted | | |  |  |
| Flutamide | Primary hepatocytes | RodgersRowland | LobellSivarajah | 17 |
| Lovastatin | pkCSM | RodgersRowland | LobellSivarajah | 49 |
| Saquinavir | Primary hepatocytes | RodgersRowland | LobellSivarajah | 20 |

**References**

Chao, P., Barminko, J., Novik, E. et al. (2009). Prediction of human hepatic clearance using an in vitro plated hepatocyte clearance model. *Drug Metab Lett 3*, 296-307. doi:10.2174/187231209790218073

Dawson, S., Stahl, S., Paul, N. et al. (2012). In vitro inhibition of the bile salt export pump correlates with risk of cholestatic drug-induced liver injury in humans. *Drug Metab Dispos 40*, 130-138. doi:10.1124/dmd.111.040758

Hafey, M. J., Houle, R., Tanis, K. Q. et al. (2020). A two-tiered in vitro approach to de-risk drug candidates for potential bile salt export pump inhibition liabilities in drug discovery. *Drug Metab Dispos 48*, 1147-1160. doi:10.1124/dmd.120.000086

Hallifax, D., Foster, J. A. and Houston, J. B. (2010). Prediction of human metabolic clearance from in vitro systems: Retrospective analysis and prospective view. *Pharm Res 27*, 2150-2161. doi:10.1007/s11095-010-0218-3

Kock, K., Ferslew, B. C., Netterberg, I. et al. (2014). Risk factors for development of cholestatic drug-induced liver injury: Inhibition of hepatic basolateral bile acid transporters multidrug resistance-associated proteins 3 and 4. *Drug Metab Dispos 42*, 665-674. doi:10.1124/dmd.113.054304

Kostrubsky, S. E., Strom, S. C., Kalgutkar, A. S. et al. (2006). Inhibition of hepatobiliary transport as a predictive method for clinical hepatotoxicity of nefazodone. *Toxicological Sciences 90*, 451-459.

Louisse, J., Alewijn, M., Peijnenburg, A. et al. (2020). Towards harmonization of test methods for in vitro hepatic clearance studies. *Toxicol In Vitro 63*, 104722. doi:10.1016/j.tiv.2019.104722

Morgan, R. E., van Staden, C. J., Chen, Y. et al. (2013). A multifactorial approach to hepatobiliary transporter assessment enables improved therapeutic compound development. *Toxicol Sci 136*, 216-241. doi:10.1093/toxsci/kft176

Wood, F. L., Houston, J. B. and Hallifax, D. (2018). Importance of the unstirred water layer and hepatocyte membrane integrity in vitro for quantification of intrinsic metabolic clearance. *Drug Metab Dispos 46*, 268-278. doi:10.1124/dmd.117.078949
